# Supplementary material for: Signatures of optimal codon usage in metabolic genes inform budding yeast ecology
Source: PLoS Biol. 2021 Apr 19;19(4):e3001185. doi: 10.1371/journal.pbio.3001185 (PMC8084343; doi:10.1371/journal.pbio.3001185)
Supplement: S1 Fig — Various features of galactose metabolism plotted on a phylogeny of the budding yeast subphylum Saccharomycotina; the 12 major clades of the subphylum are color coded. The presence and codon optimization (measured by estAI) of the 3 GAL genes are represented in the inner 3 rings. The GAL clusters in the Dipodascaceae/Trichomonascaceae, Pichiaceae, and Phaffomycetaceae were recently identified as likely originating from horizontal gene transfer events from the CUG-Ser1 clade. High codon optimization (darker colors) in the GAL pathway is not restricted to any one major clade. Complete and clustered occurrences of the GAL pathway are represented by filled-in blue squares and circles, respectively. Ecological associations were uncovered using a literature search (S2 Data). (PDF) [file pbio.3001185.s001.pdf]

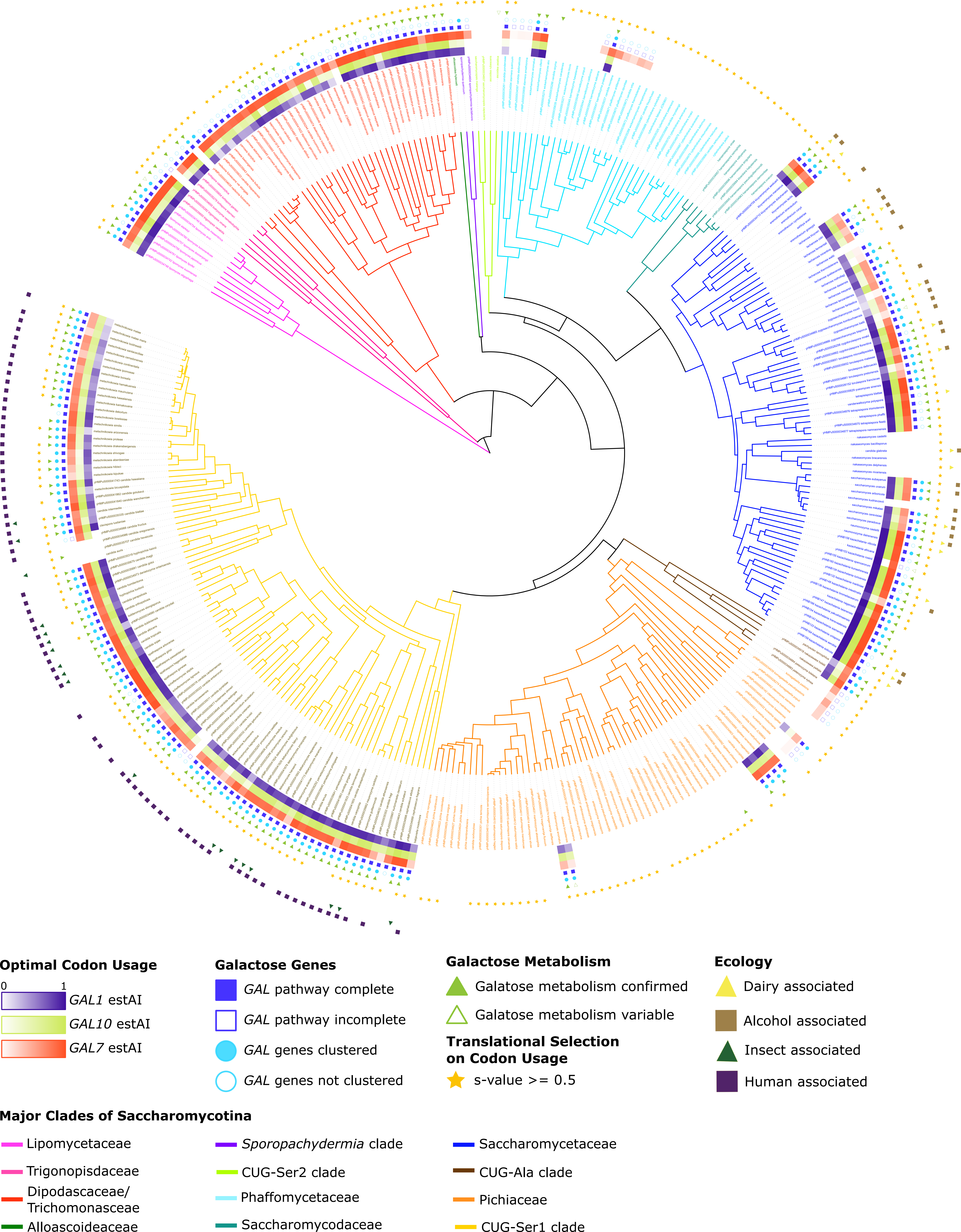

Optimal Codon Usage
0
1
GAL1 estAI
GAL10 estAI
GAL7 estAI
Galactose Genes
GAL pathway complete
GAL pathway incomplete
GAL genes clustered
GAL genes not clustered
Galactose Metabolism
Galatose metabolism confirmed
Galatose metabolism variable
Translational Selection on Codon Usage
s-value >= 0.5
Ecology
Dairy associated
Alcohol associated
Insect associated
Human associated
Major Clades of Saccharomycotina
Lipomycetaceae
Trigonopisdaceae
Dipodascaceae/ Trichomonasceae
Alloascoideaceae
Sporopachydermia clade
CUG-Ser2 clade
Phaffomycetaceae
Saccharomycodaceae
Saccharomycetaceae
CUG-Ala clade
Pichiaceae
CUG-Ser1 clade
