# Supplementary figures and images for: Signatures of optimal codon usage in metabolic genes inform budding yeast ecology
Source: PLoS Biol. 2021 Apr 19;19(4):e3001185. doi: 10.1371/journal.pbio.3001185 (PMC8084343; doi:10.1371/journal.pbio.3001185)

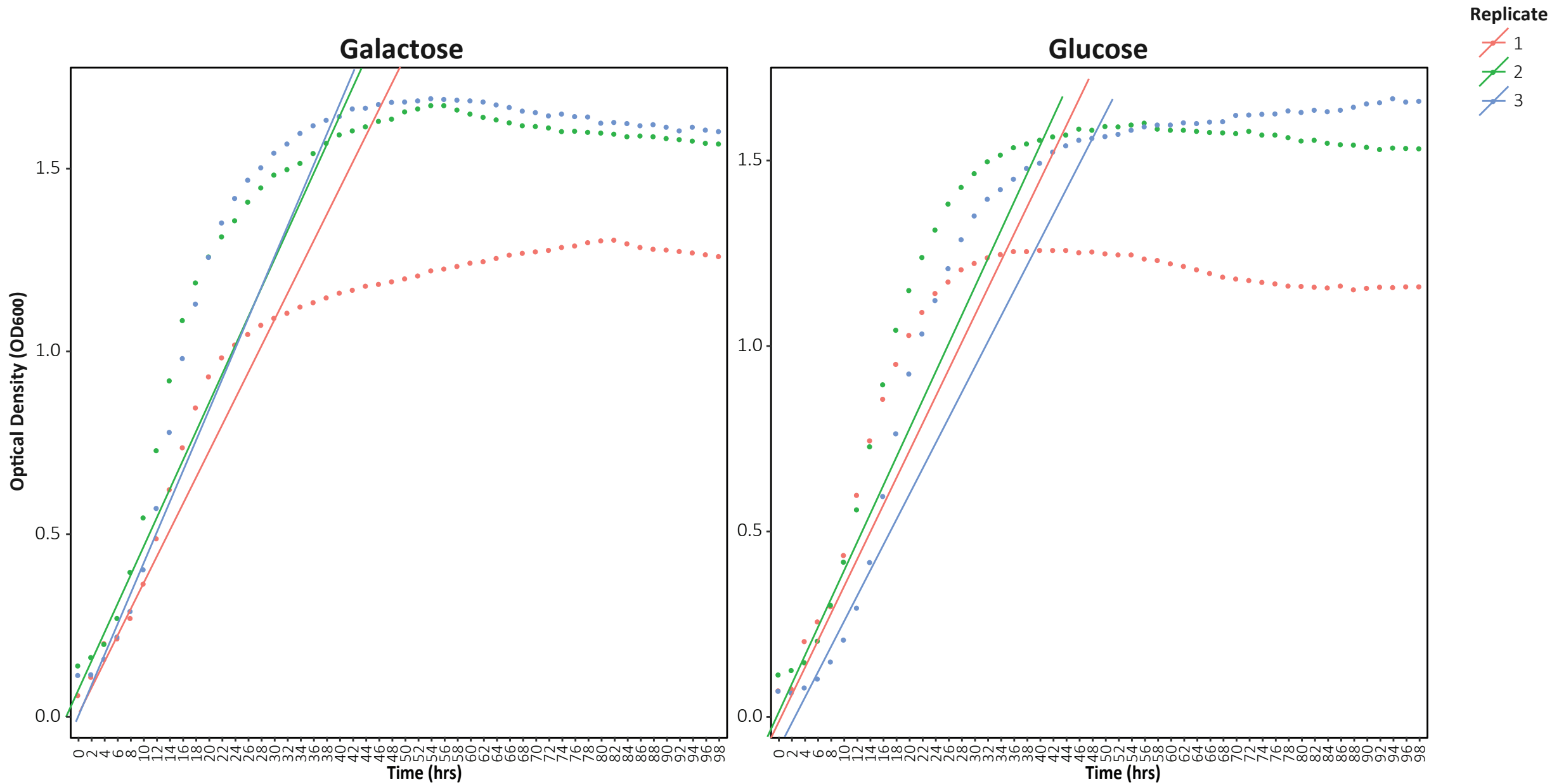

Supplement: S2 Fig — Growth rate is calculated based on the maximum slope of the curve in the R package grofit. The slopes calculated in this species for galactose are 0.0495, 0.0747, and 0.0862 in the replicates 1 to 3 for an average growth rate of 0.070. The slopes calculated in this species for glucose are 0.0712, 0.0762, and 0.0682 in the replicates 1 to 3 for an average growth rate of 0.072. Therefore, the glucose normalized rate of growth on galactose is 0.97. (PDF) [file pbio.3001185.s002.pdf]

A.

*GAL1*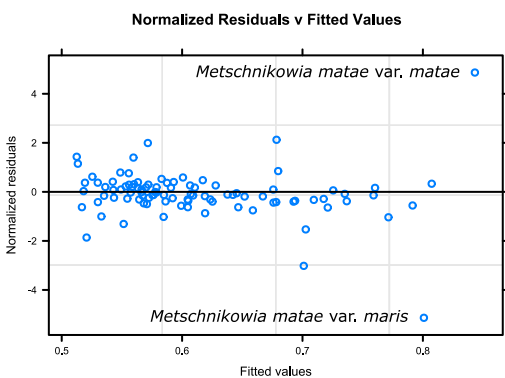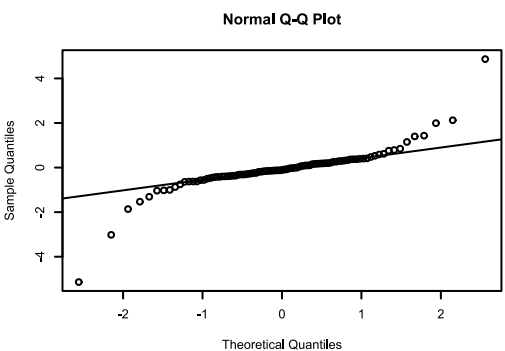*GAL10*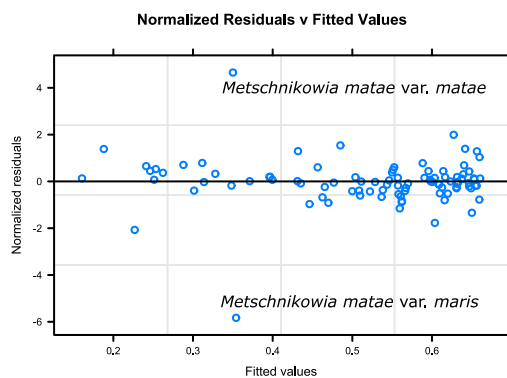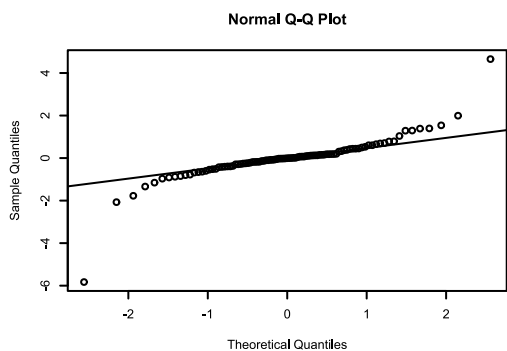*GAL7*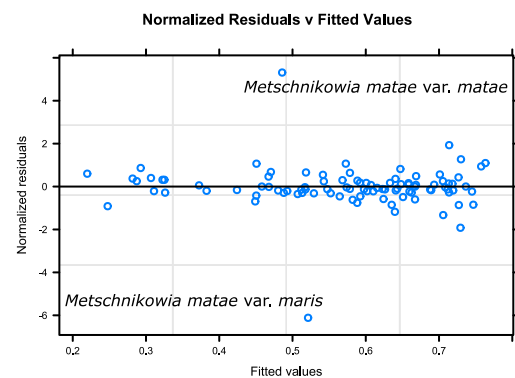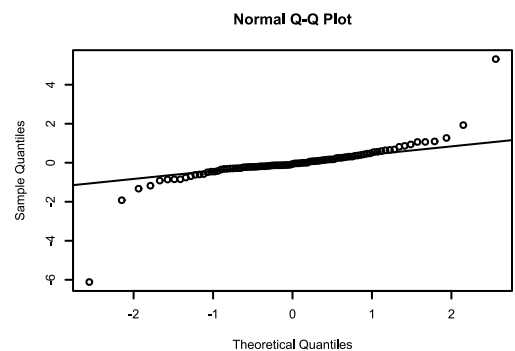

B.

*GAL1*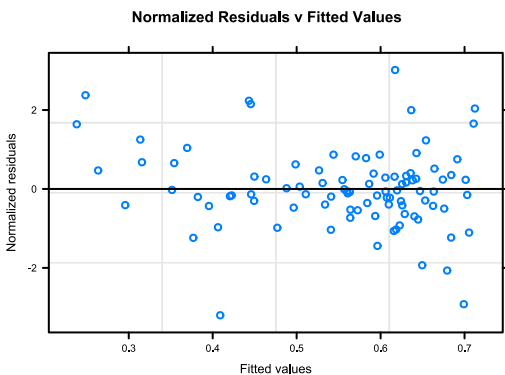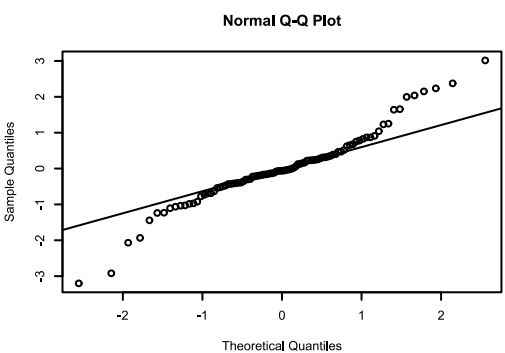*GAL10*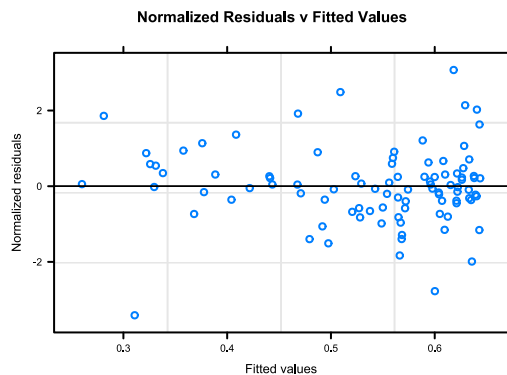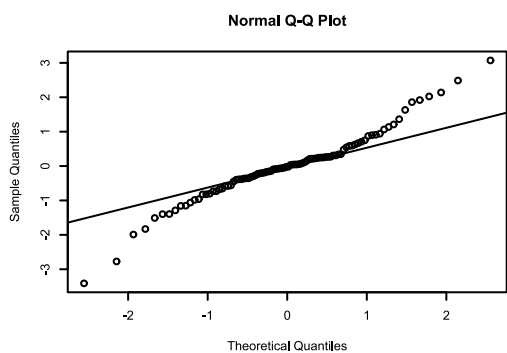*GAL7*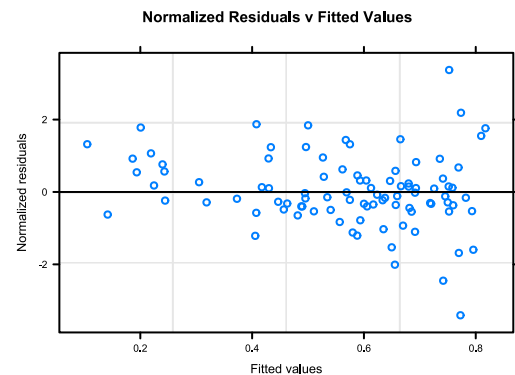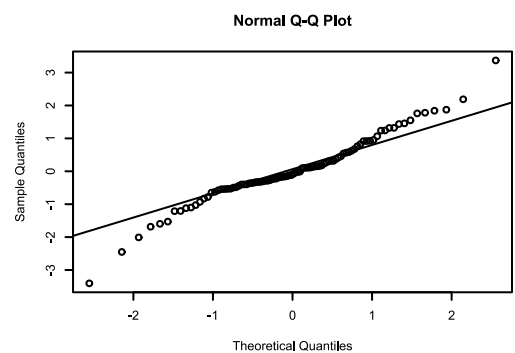

Supplement: S3 Fig — (A) The residual versus fitted analysis shows 2 outlier strains: Metschnikowia matae var. matae and Metschnikowia matae var. maris. (B) Residual and Q-Q normalized plots after removal of Metschnikowia matae var. matae. No clear outliers remained after removal of this species. (PDF) [file pbio.3001185.s003.pdf]

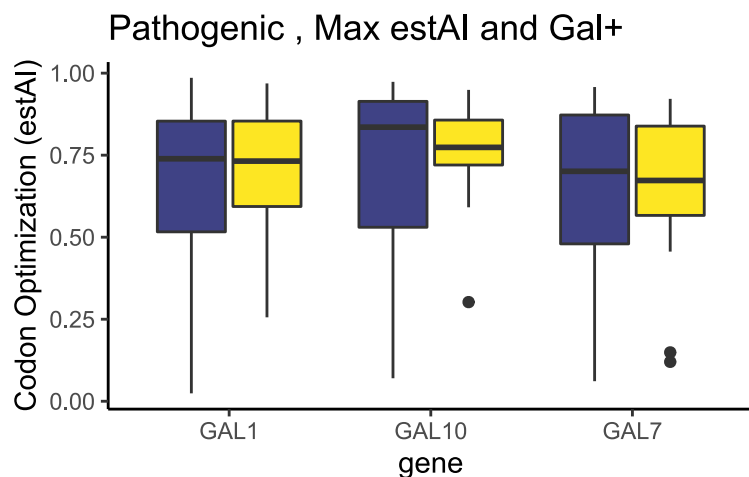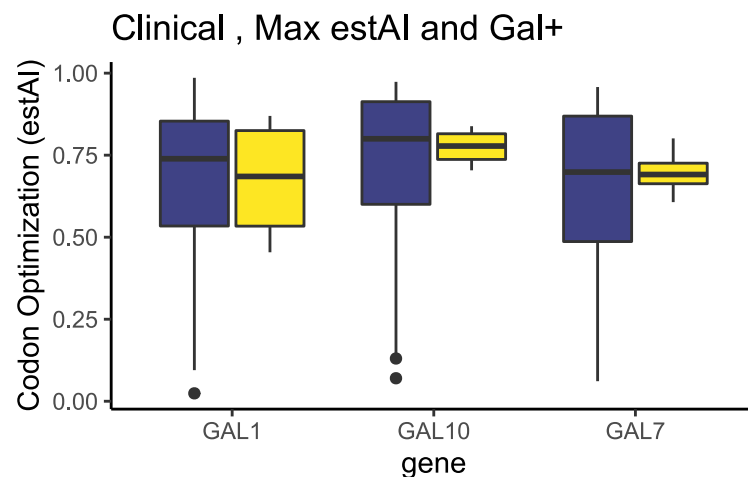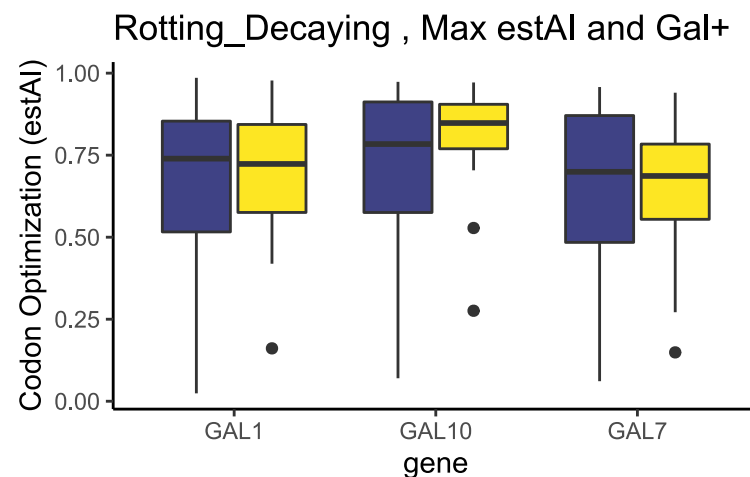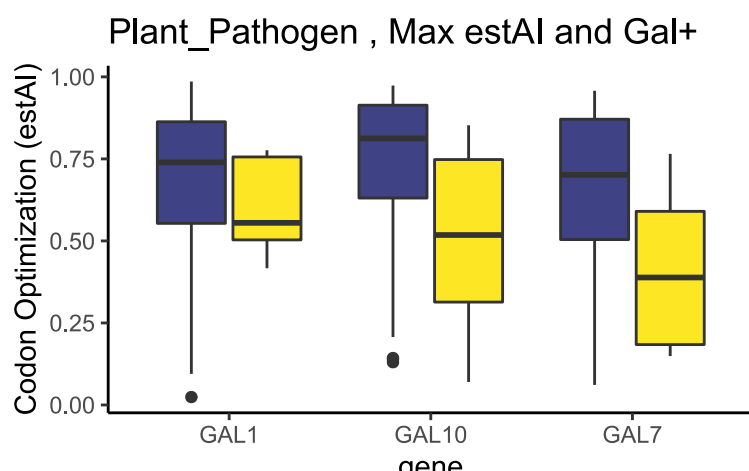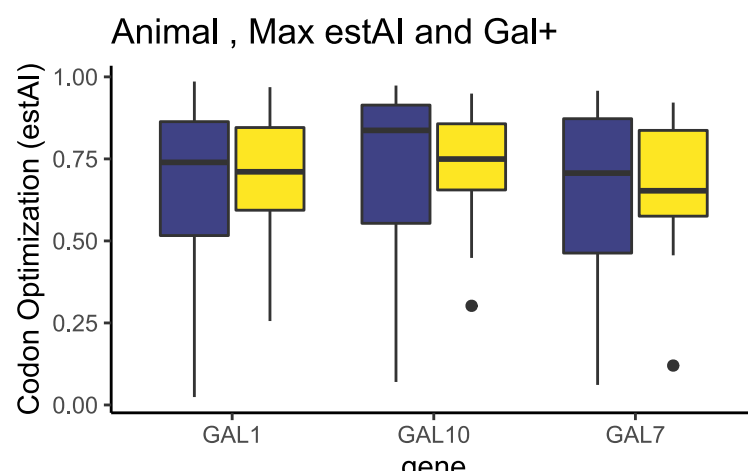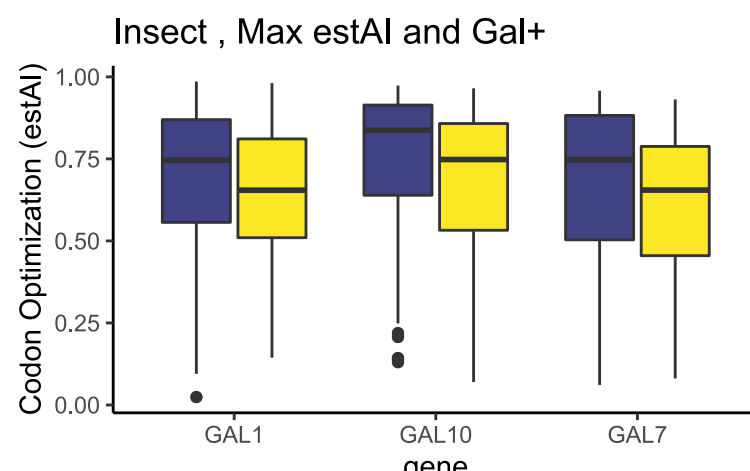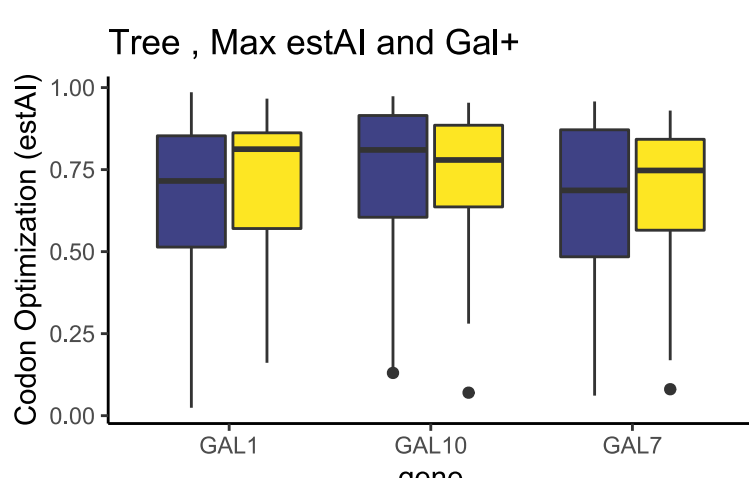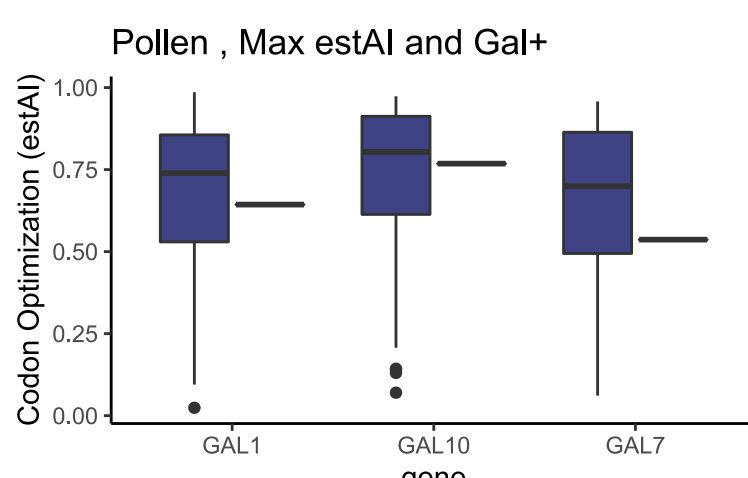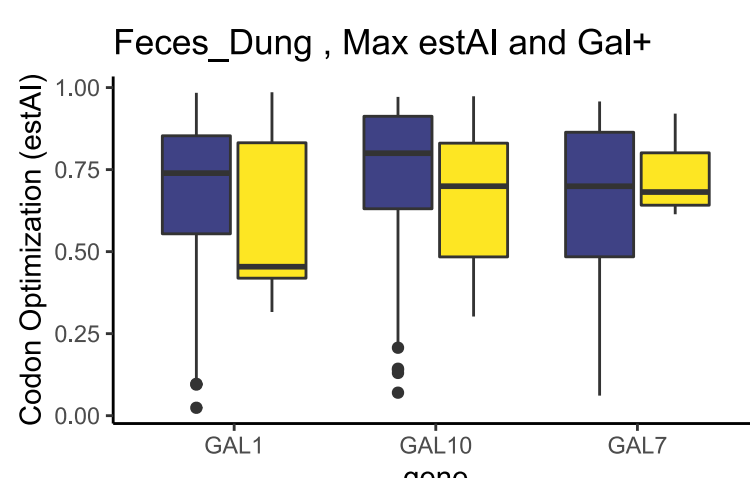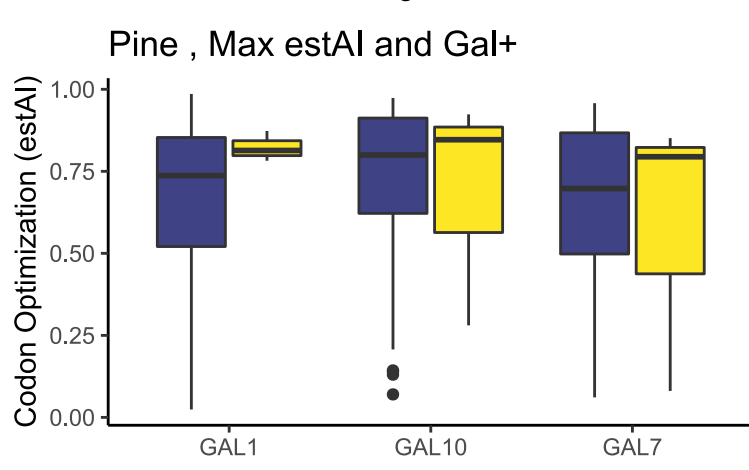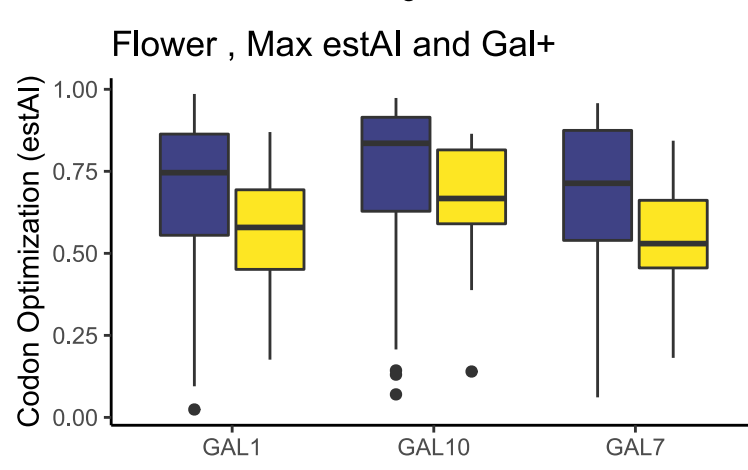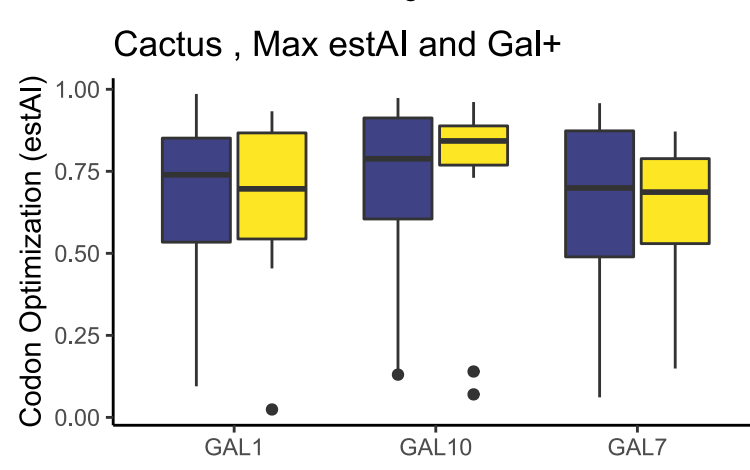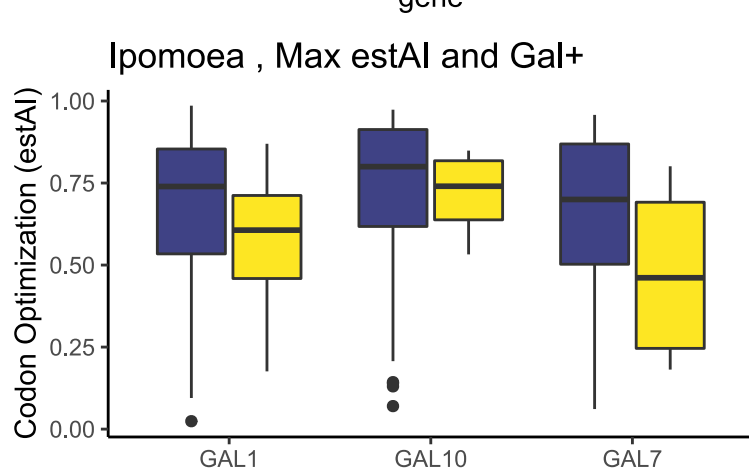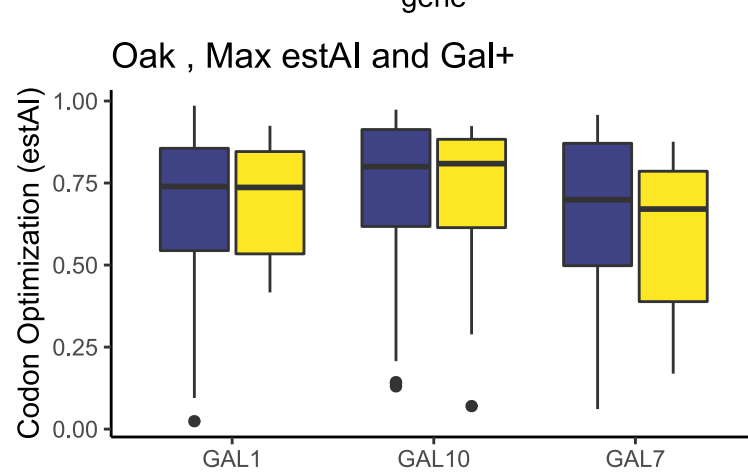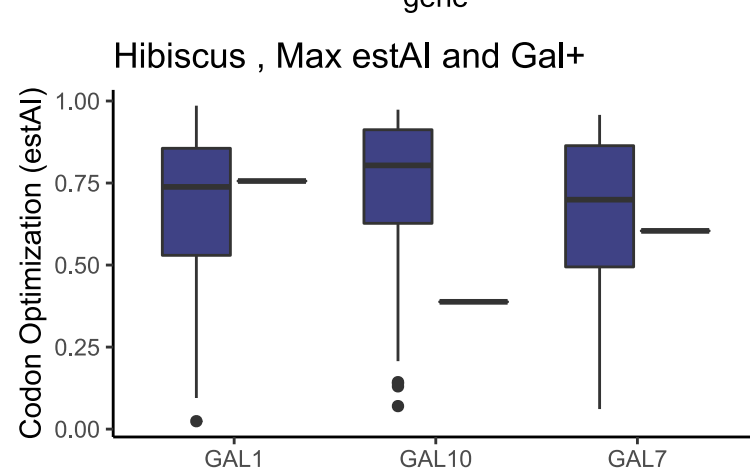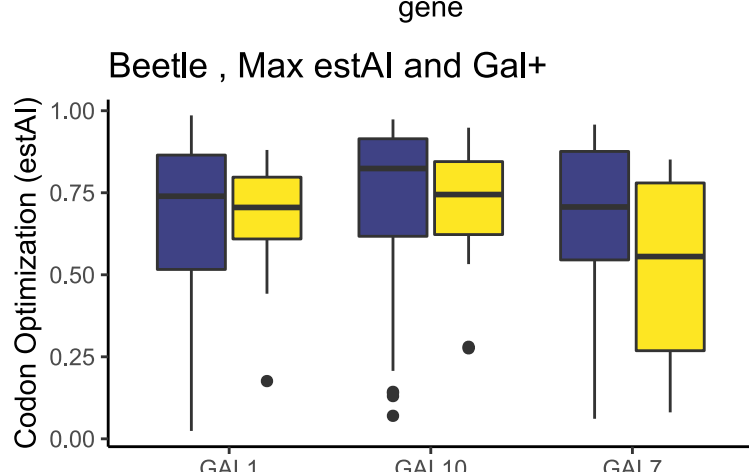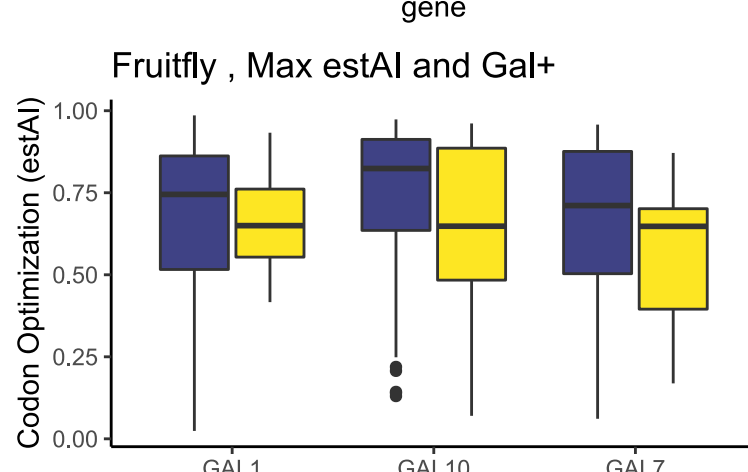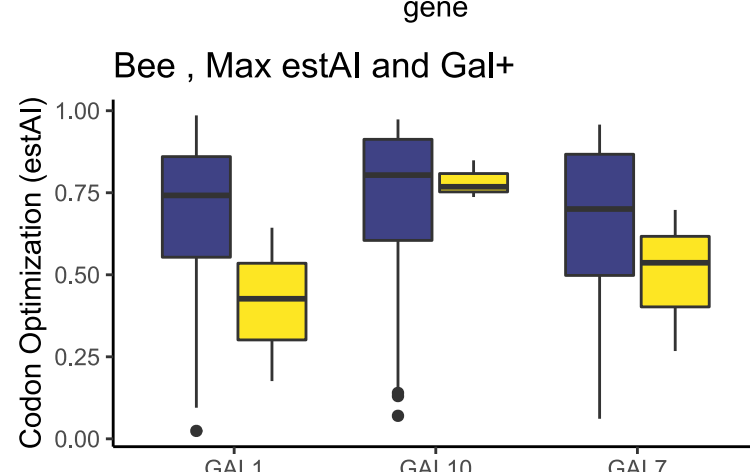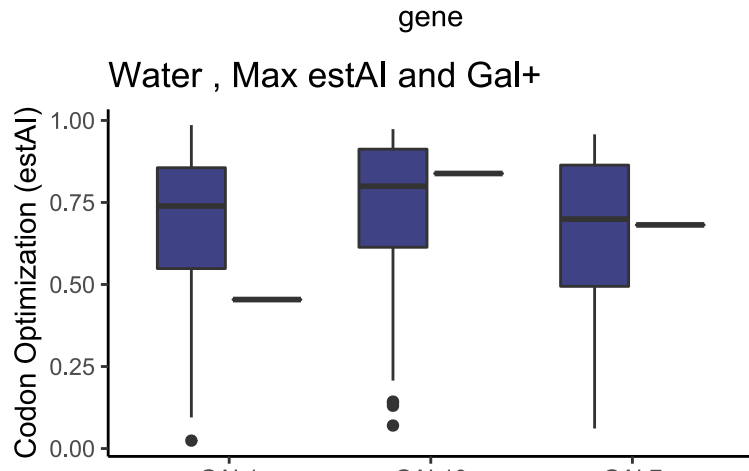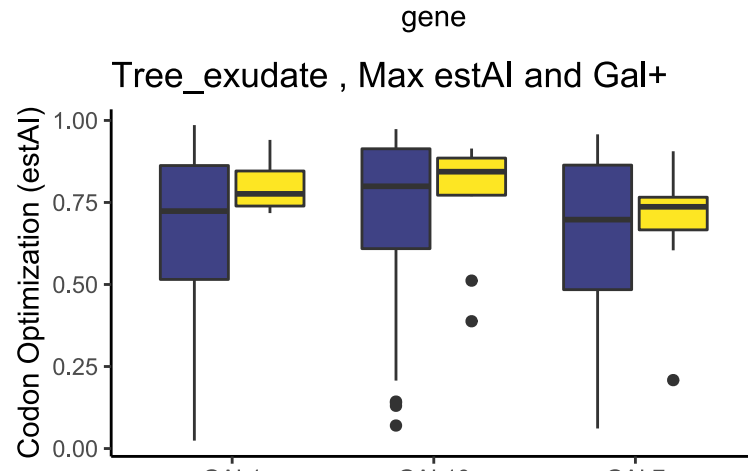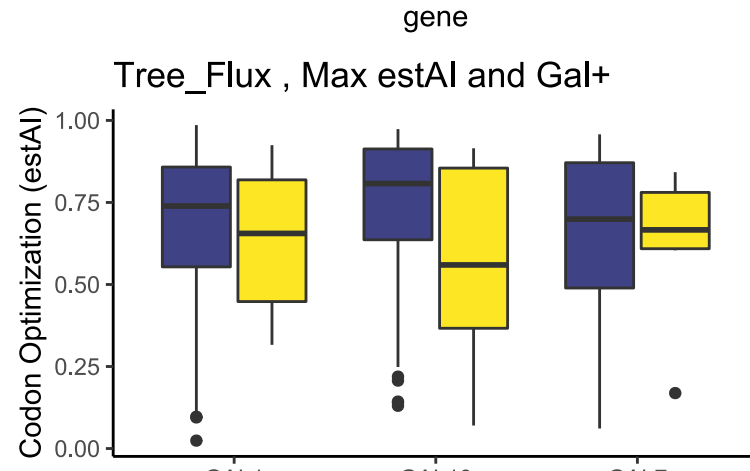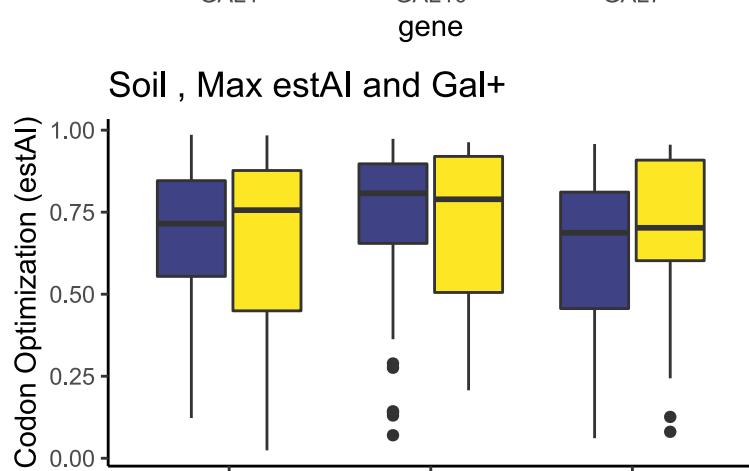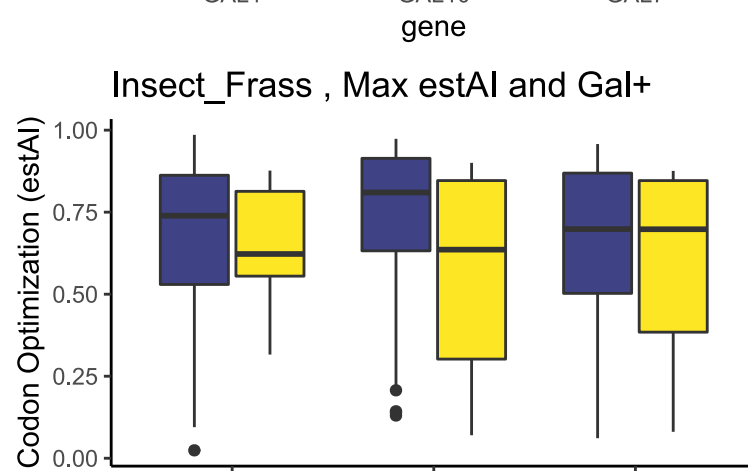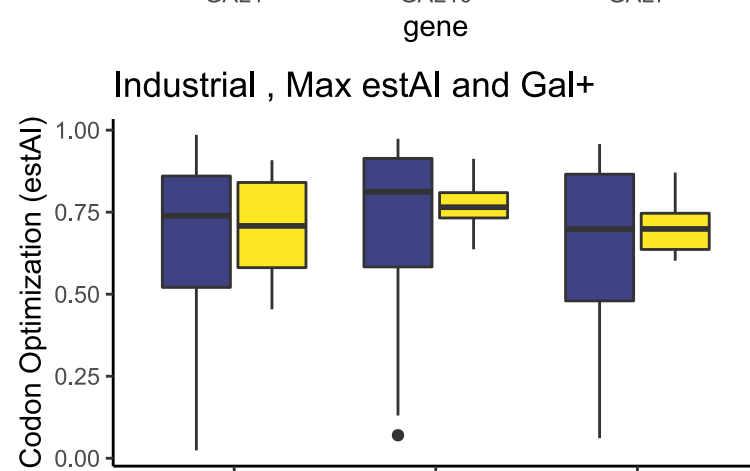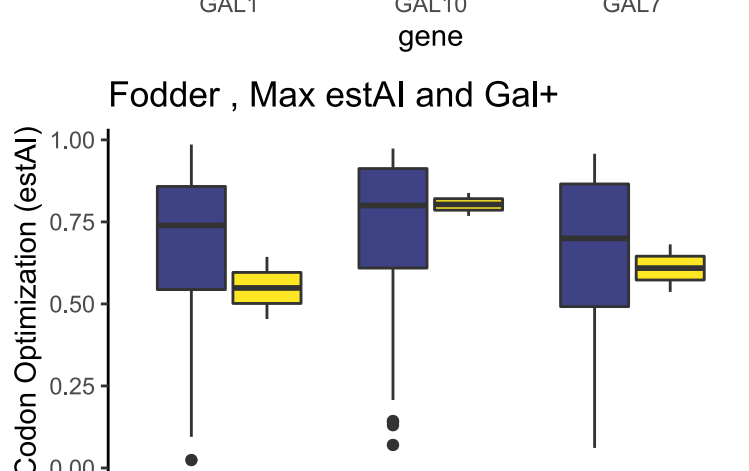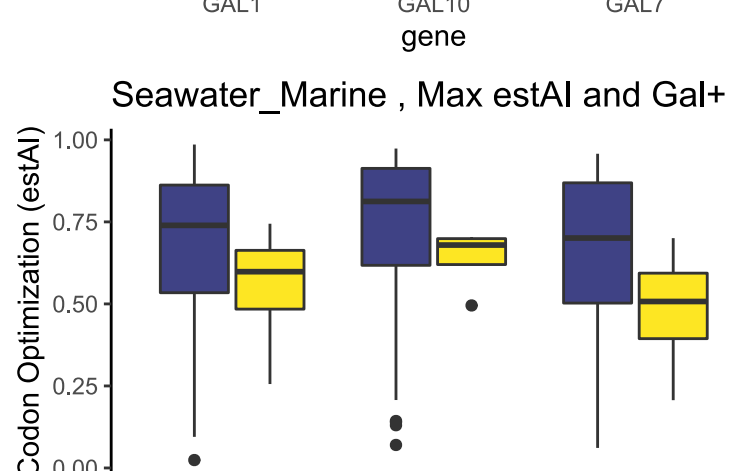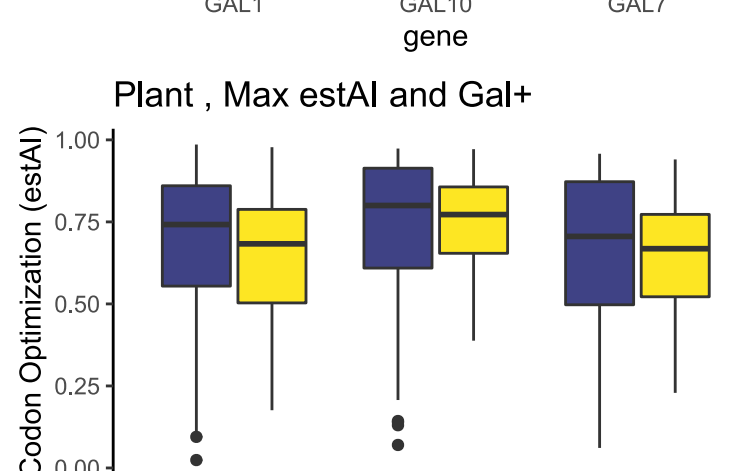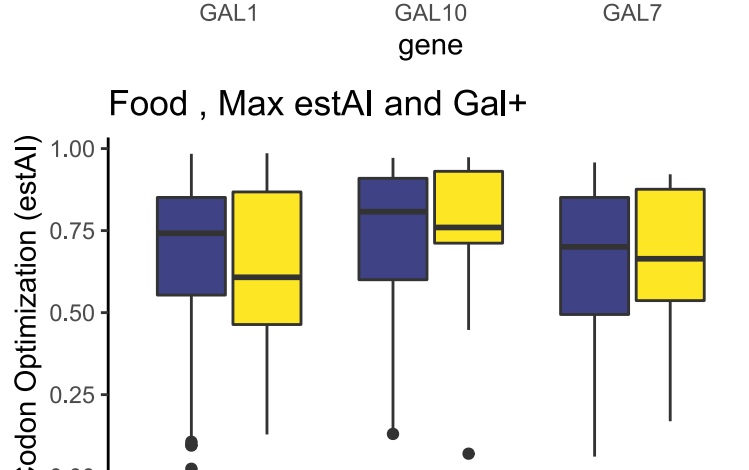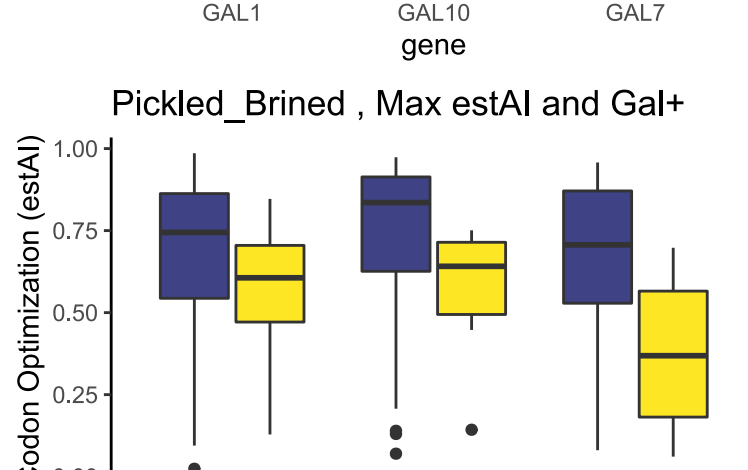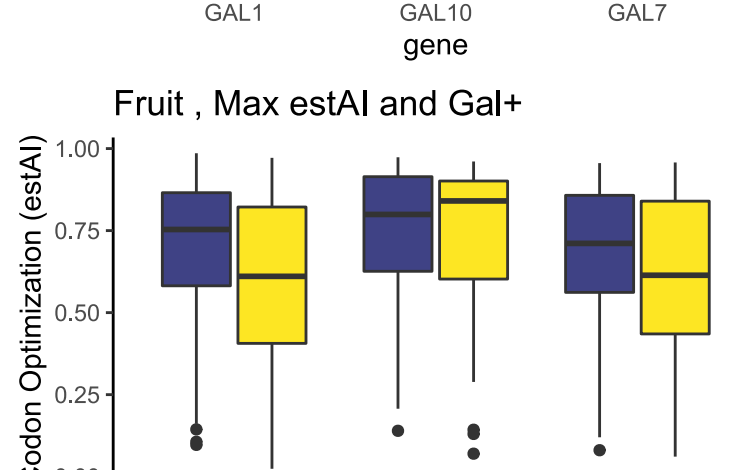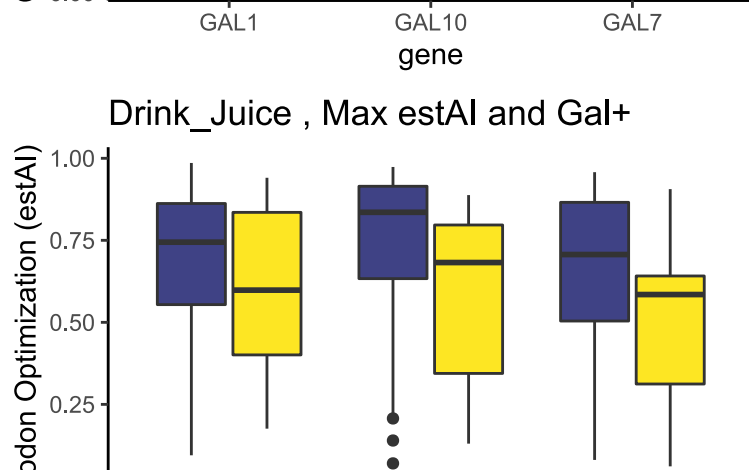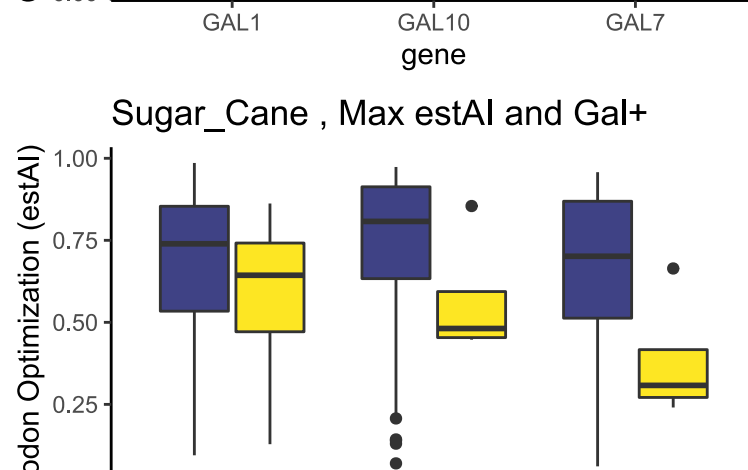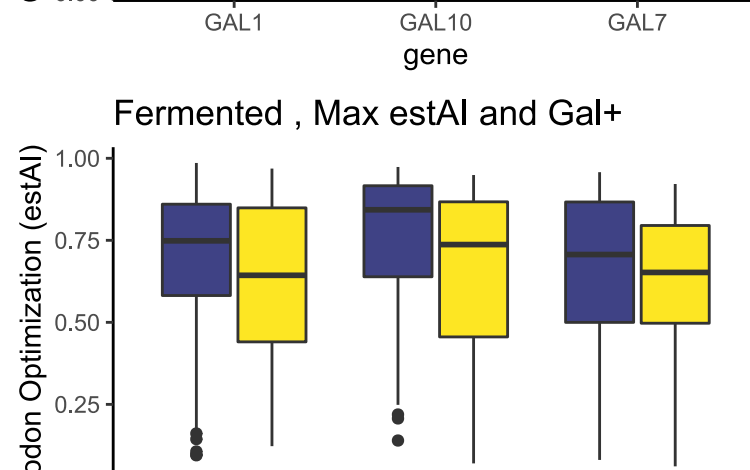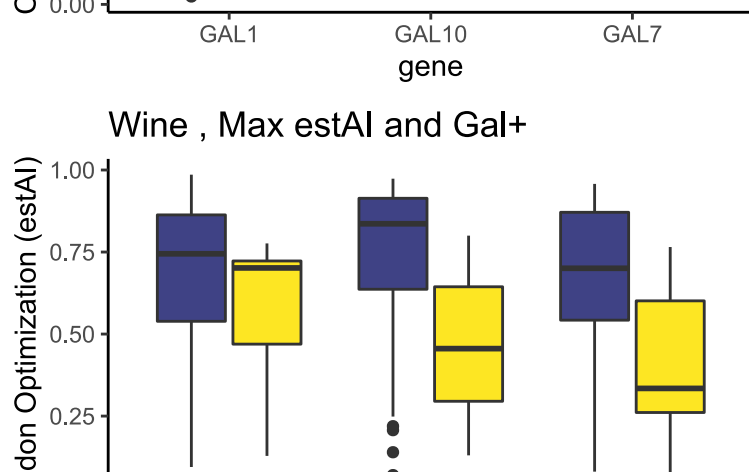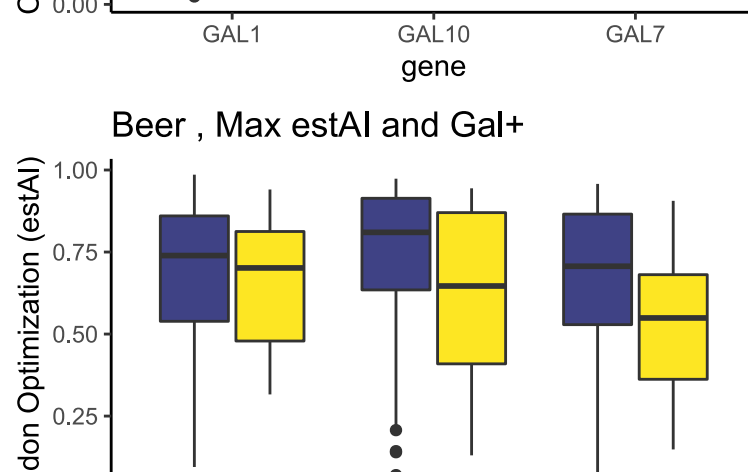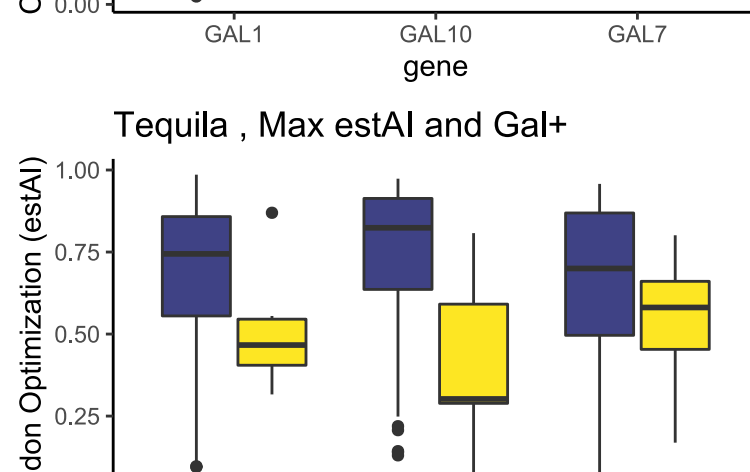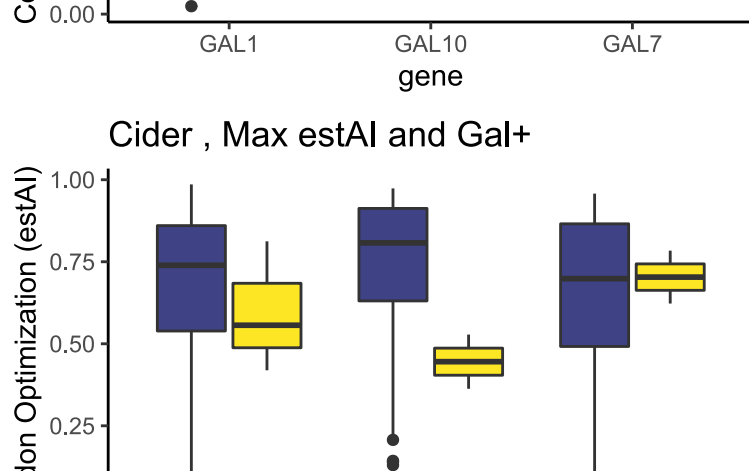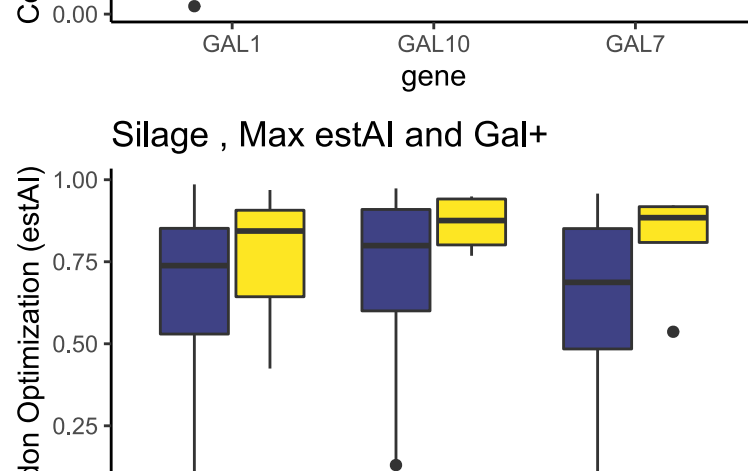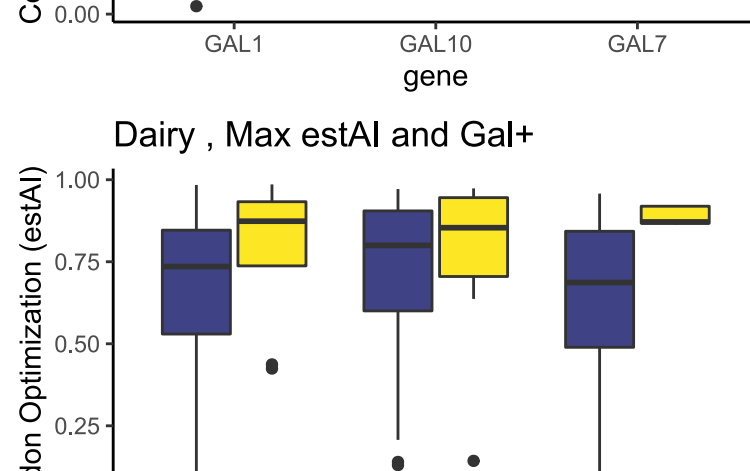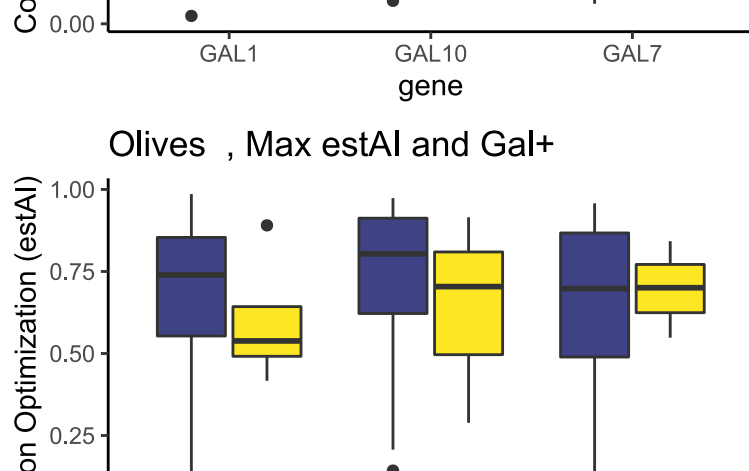

Supplement: S4 Fig — Blue bars are the codon optimization values for species that have not been isolated from the particular ecology. Yellow bars are the codon optimization values for species that have been isolated from that ecology. Ecological information was tested in 50 isolation environments from data collated from The Yeasts: A Taxonomic Study as recorded by Opulente and colleagues [54]. (PDF) [file pbio.3001185.s004.pdf]

A

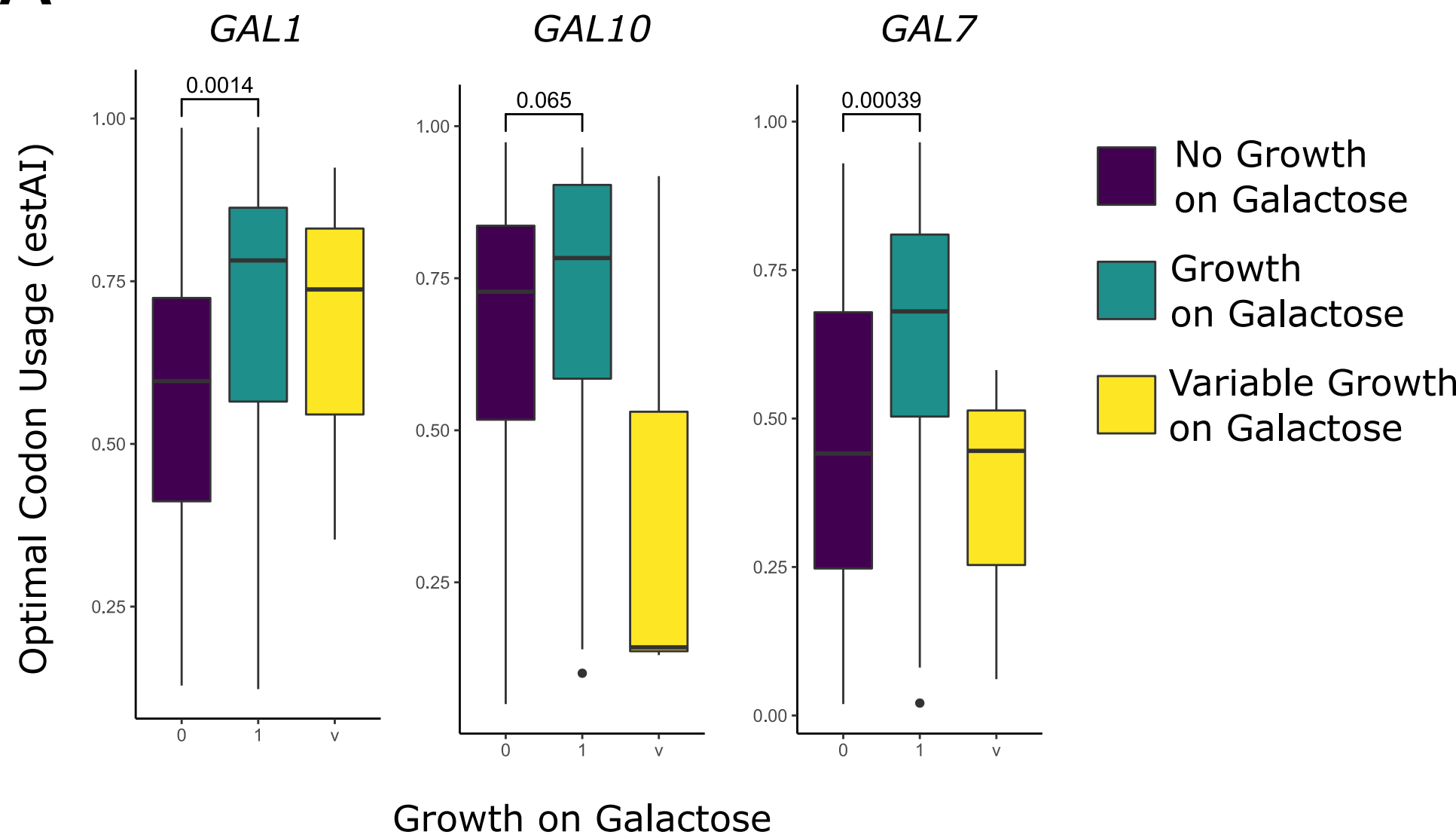

B

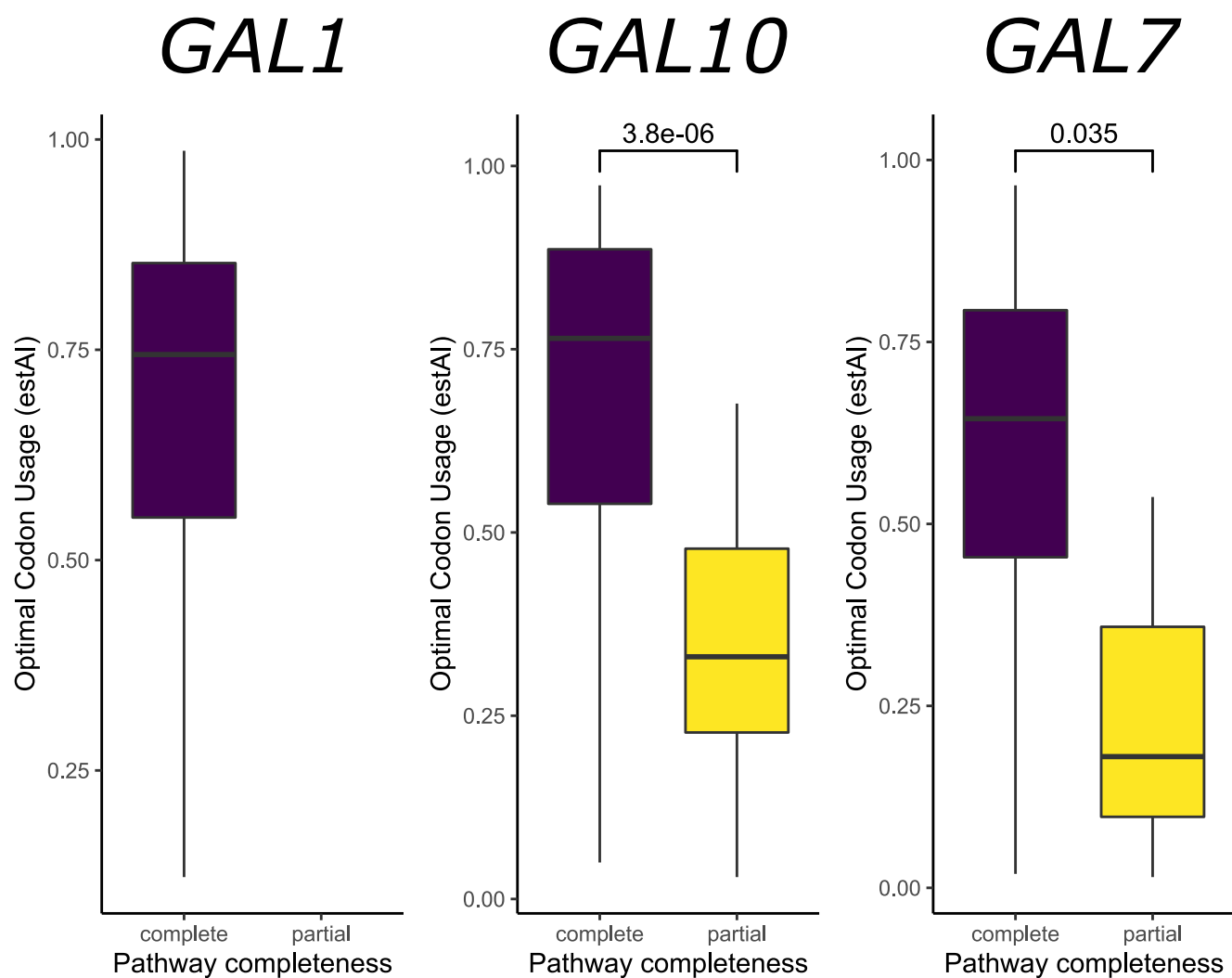

Supplement: S5 Fig — (A) Wilcoxon rank sum test of GAL codon optimization versus binary data for growth on galactose. A total of 170 species were included in this analysis. (B) Wilcoxon rank sum test of GAL codon optimization in species with complete or incomplete GAL pathways. A total of 185 species were included in this analysis. (PDF) [file pbio.3001185.s005.pdf]

# A. Saccharomycetaceae

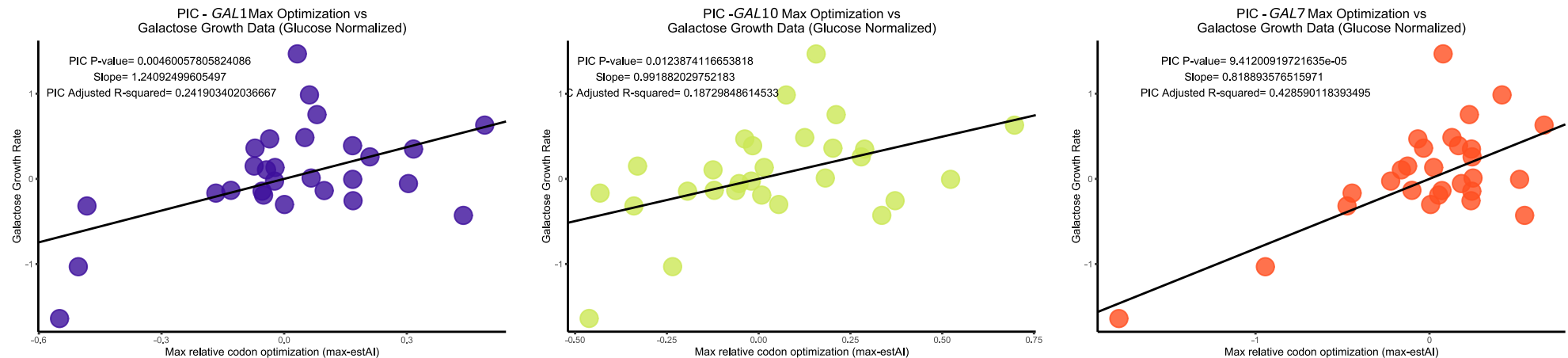

# B. CUG-Ser1 Clade

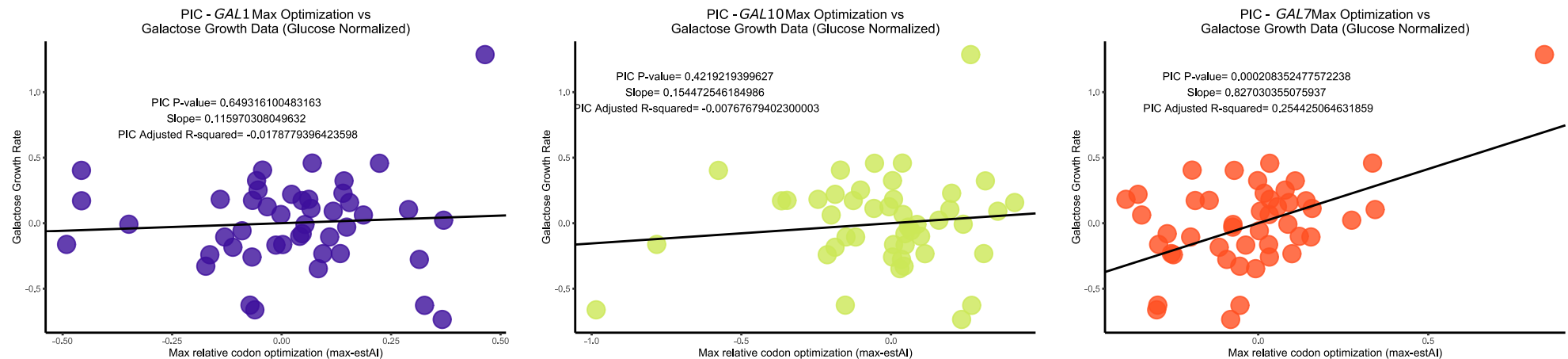

Supplement: S6 Fig — (A) The phylogenetically corrected correlation between GAL codon optimization and quantitative growth on galactose-containing medium is significant in all genes when only species from the family Saccharomycetaceae are considered. A total of 29 species were included in this analysis. (B) The phylogenetically corrected correlation between GAL codon optimization and quantitative growth on galactose-containing medium is only significant in GAL7 when only the CUG-Ser1 major clade species are considered. This analysis includes 47 species. (PDF) [file pbio.3001185.s006.pdf]

# PIC - *PGM1/2* Max Optimization vs Galactose Growth Data (Glucose Normalized)

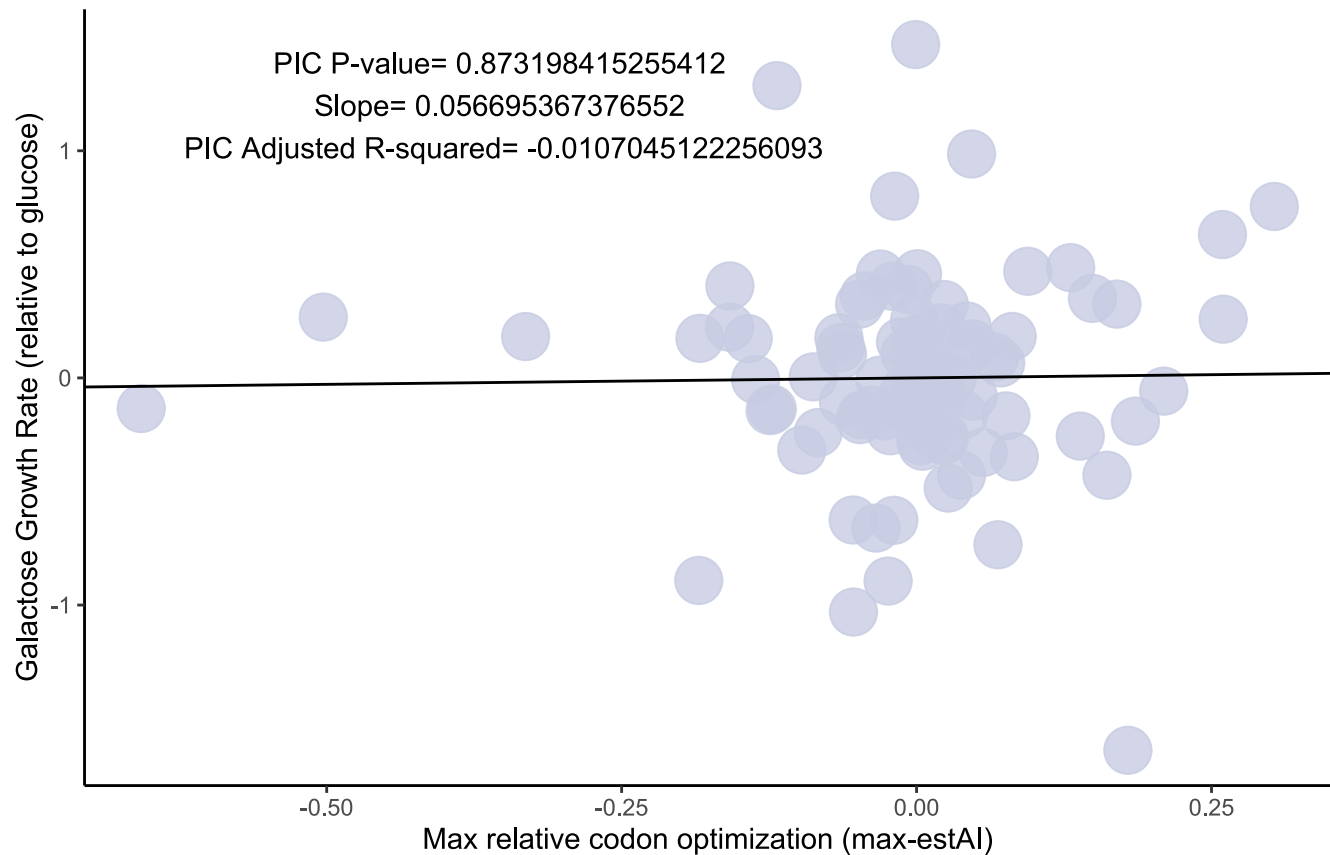

Supplement: S7 Fig — This analysis suggests that codon optimization in PGM1/PGM2 does not contribute to growth on galactose-containing medium. (PDF) [file pbio.3001185.s007.pdf]

PIC - PMI40 Codon Optimization vs  
Galactose Growth (Glucose Normalized)  
With S-value Filter

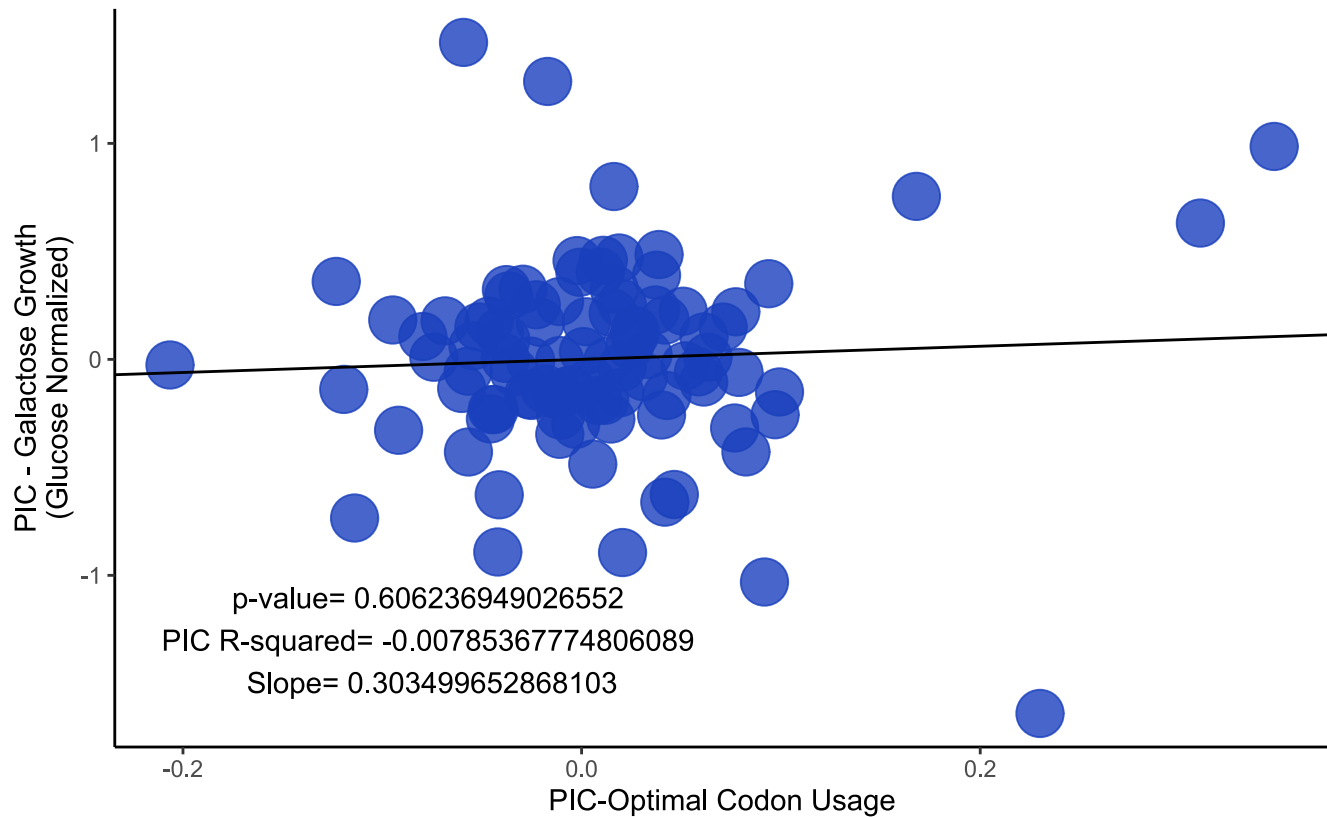

Supplement: S8 Fig — (PDF) [file pbio.3001185.s008.pdf]

A

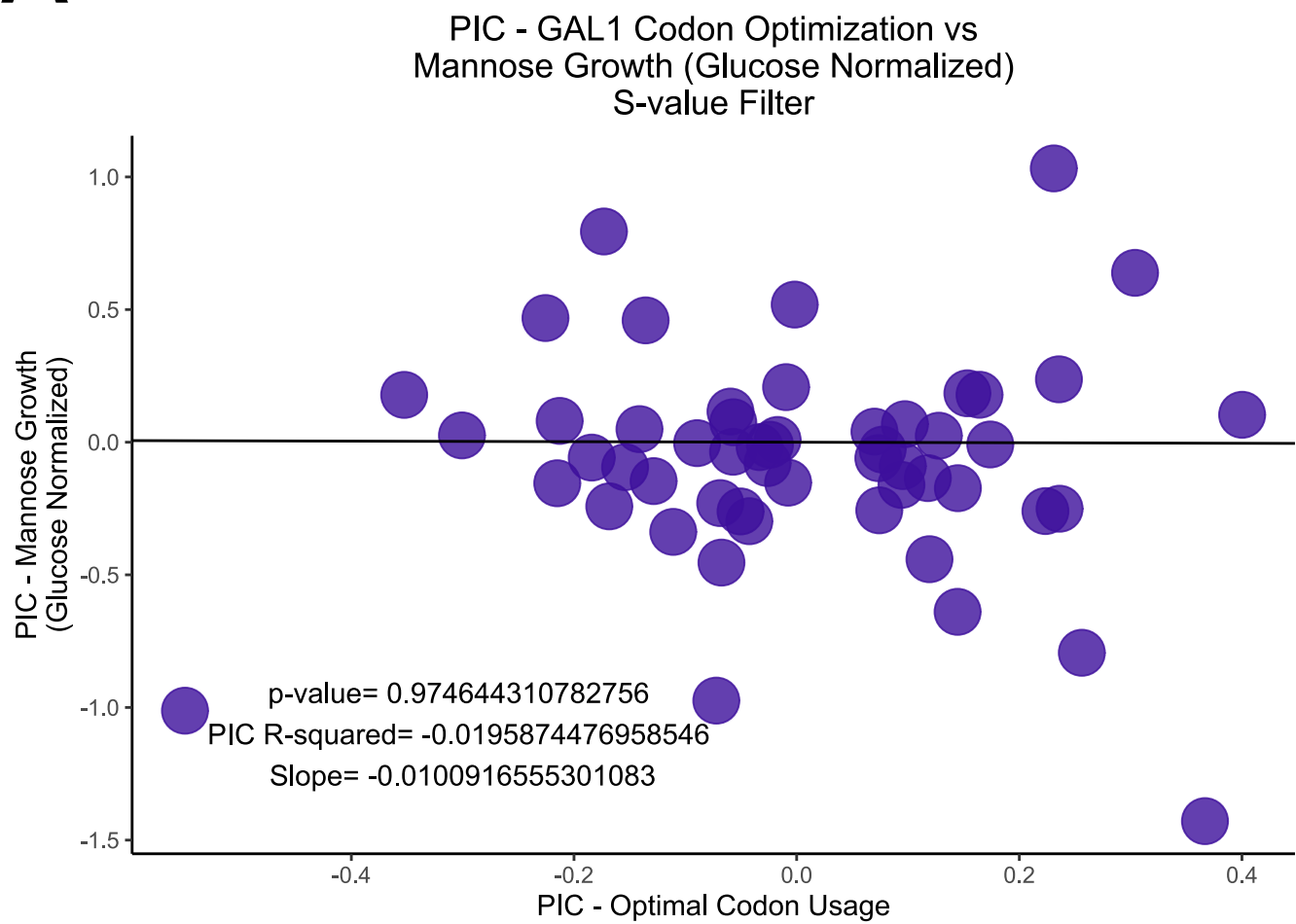

B

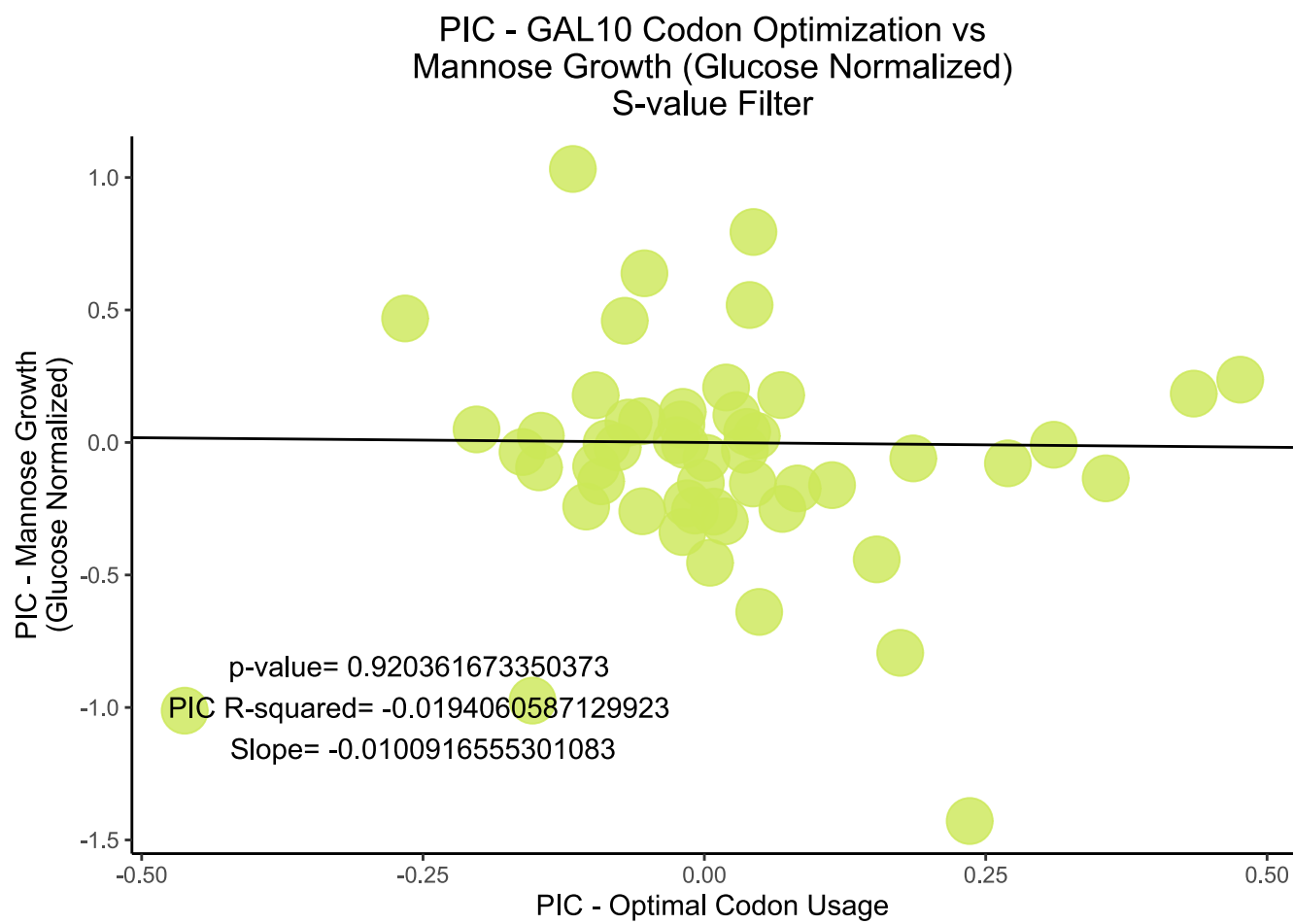

C

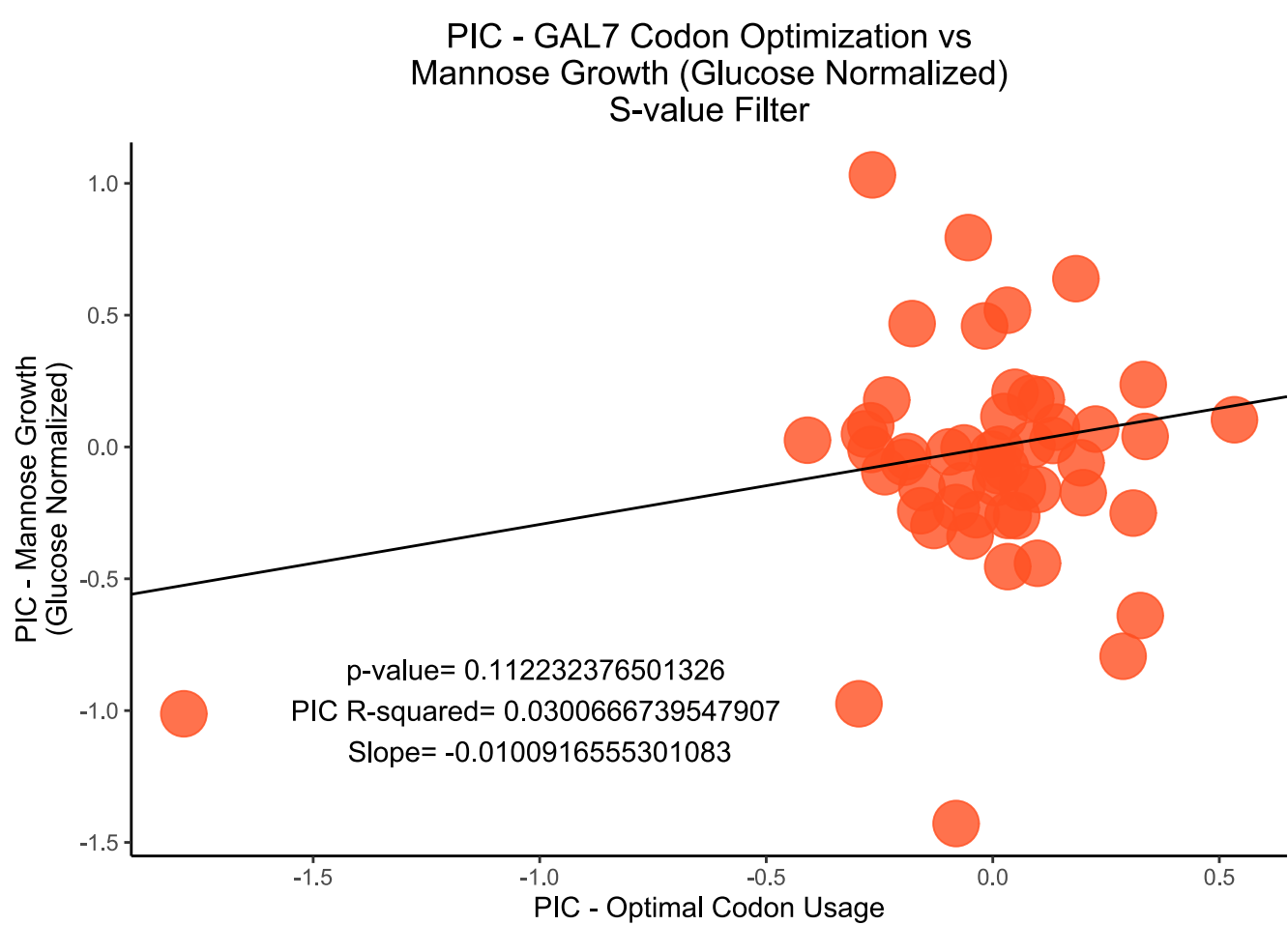

Supplement: S9 Fig — This result supports the conclusion that the association between GAL optimization and growth rate on galactose is specific to that pathway. (PDF) [file pbio.3001185.s009.pdf]

PIC - PMI40 Codon Optimization vs  
Galactose Growth (Glucose Normalized)  
With S-value Filter

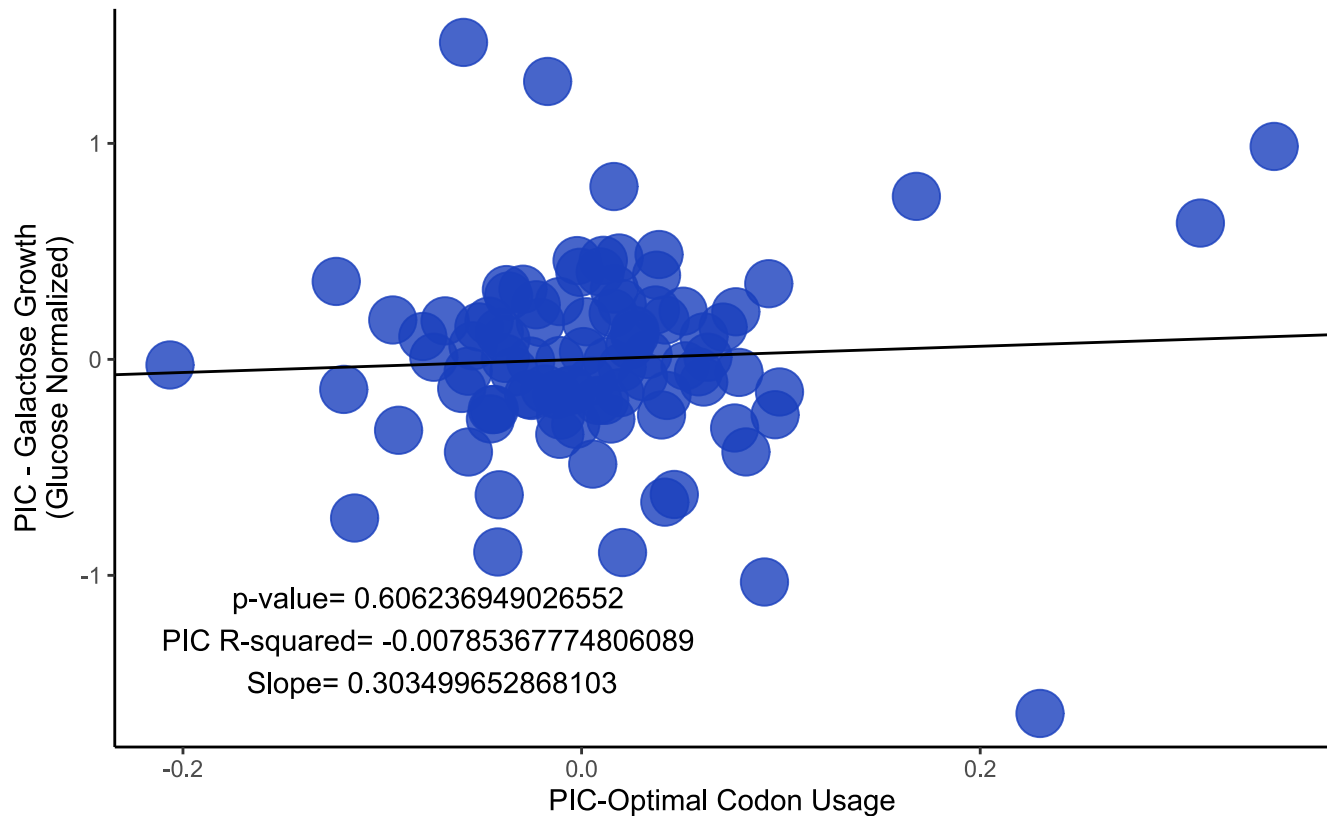

Supplement: S10 Fig — This result is likely due to the very high codon optimization observed in PMI40. (PDF) [file pbio.3001185.s010.pdf]

## A. *Kluyveromyces wickerhamii*

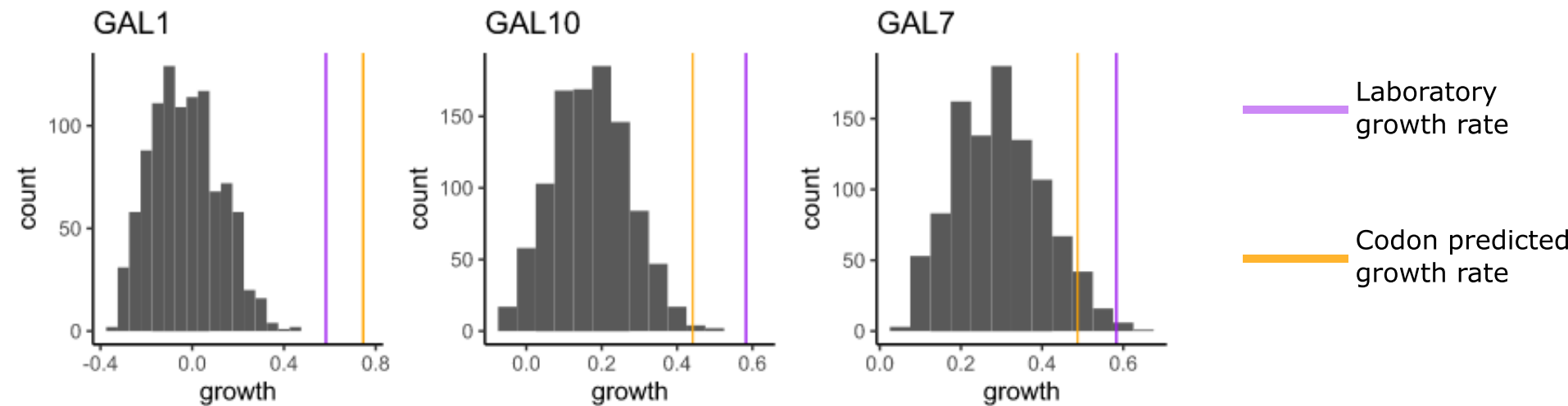

## B. *Wickerhamiella occidentalis*

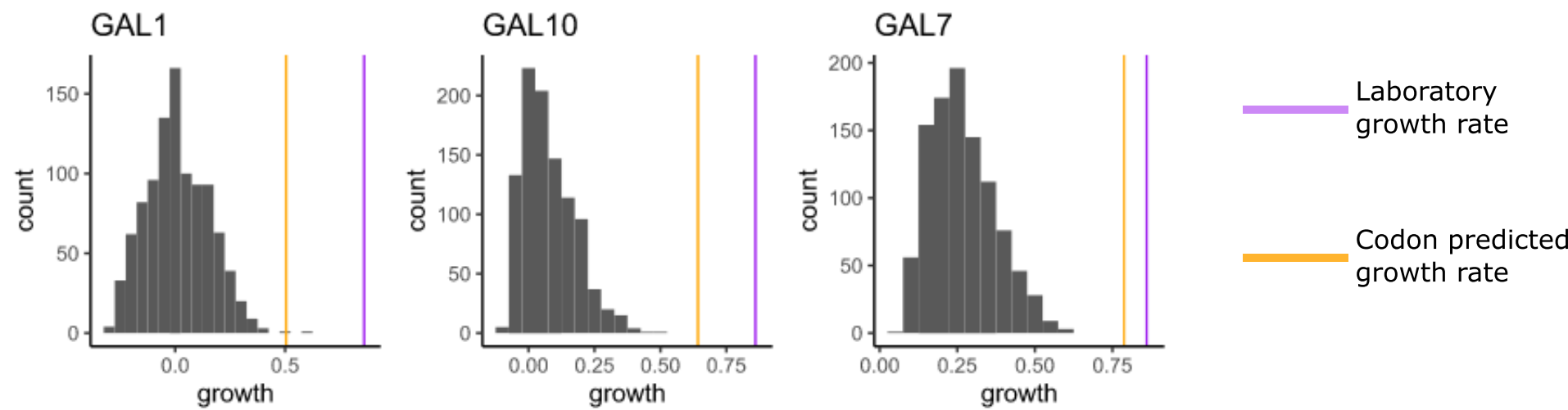

Supplement: S12 Fig — To better understand the performance of our growth rate predictions based on codon usage, we tested how well GALactose genes with randomly assigned codons could predict growth rate. The purple lines represent the empirically measured growth rate for each species. The orange line is the growth rate predicted by the actual codon usage for each gene based on the PGLS analysis. For each GAL gene associated with K. wickerhamii (panel A) and W. occidentalis (panel B), we generated 1,000 DNA sequences with random codon usage (while keeping the protein sequence identical). For each random sample, we then predicted growth rate based on our regression analysis. In both cases, both the predicted and the actual growth rates fall outside of the 95th percentile. Additionally, for all but one observation (GAL7 in K. wickerhamii), the predictions and actual growth rate fall outside the 99th percentile. These results show that our predictions are highly informative. (PDF) [file pbio.3001185.s012.pdf]
