# Supplementary material for: Signatures of optimal codon usage in metabolic genes inform budding yeast ecology
Source: PLoS Biol. 2021 Apr 19;19(4):e3001185. doi: 10.1371/journal.pbio.3001185 (PMC8084343; doi:10.1371/journal.pbio.3001185)
Supplement: S11 Fig — The circular points represent the predicted growth rates based on the observed codon optimization values (lines). The triangles represent the actual growth rate measured in the laboratory. (PDF) [file pbio.3001185.s011.pdf]

A

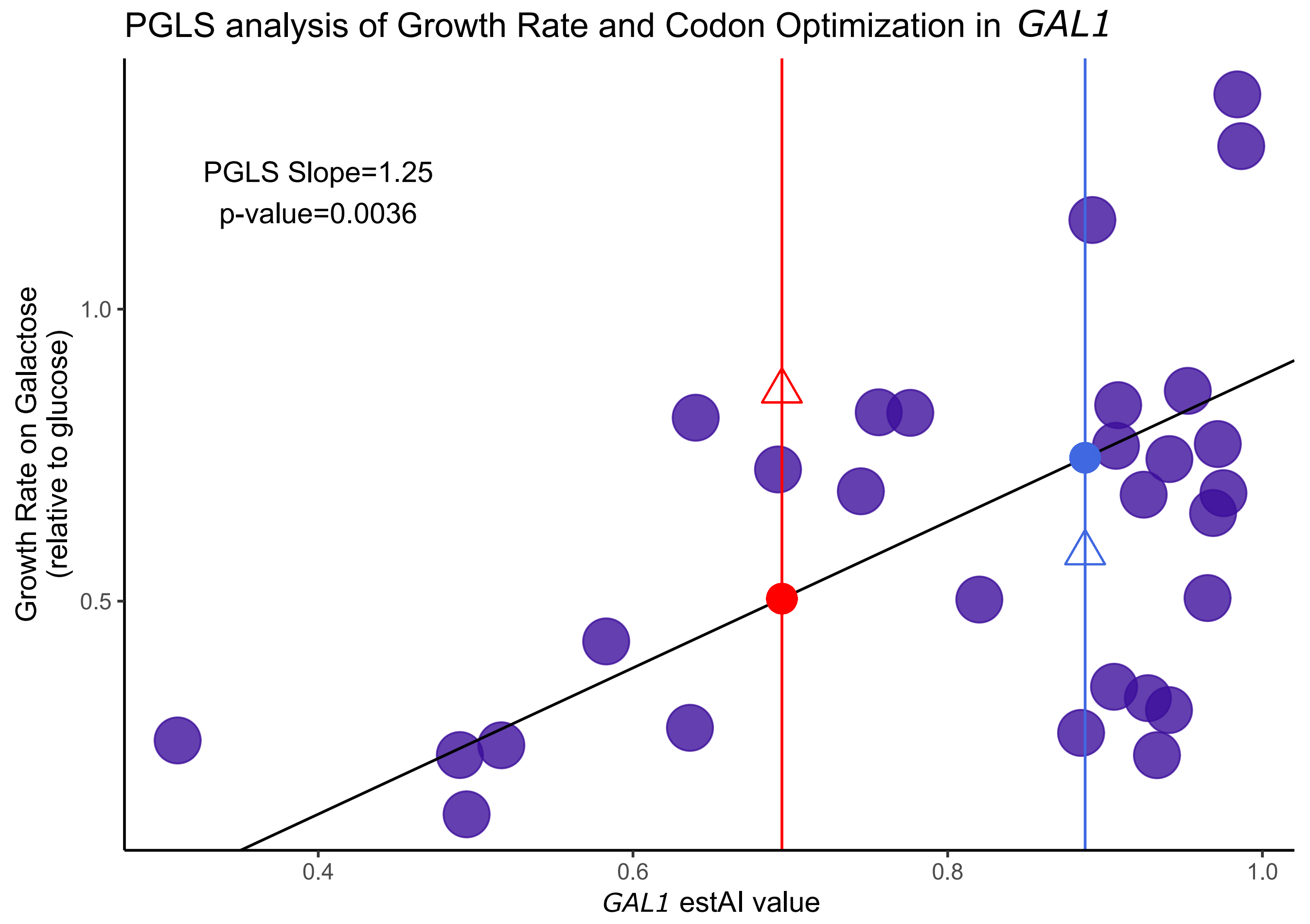
PGLS analysis of Growth Rate and Codon Optimization in GAL1
PGLS Slope=1.25
p-value=0.0036
Growth Rate on Galactose
(relative to glucose)
1.0
0.5
0.4
0.6
0.8
1.0
GAL1 estAI value

B

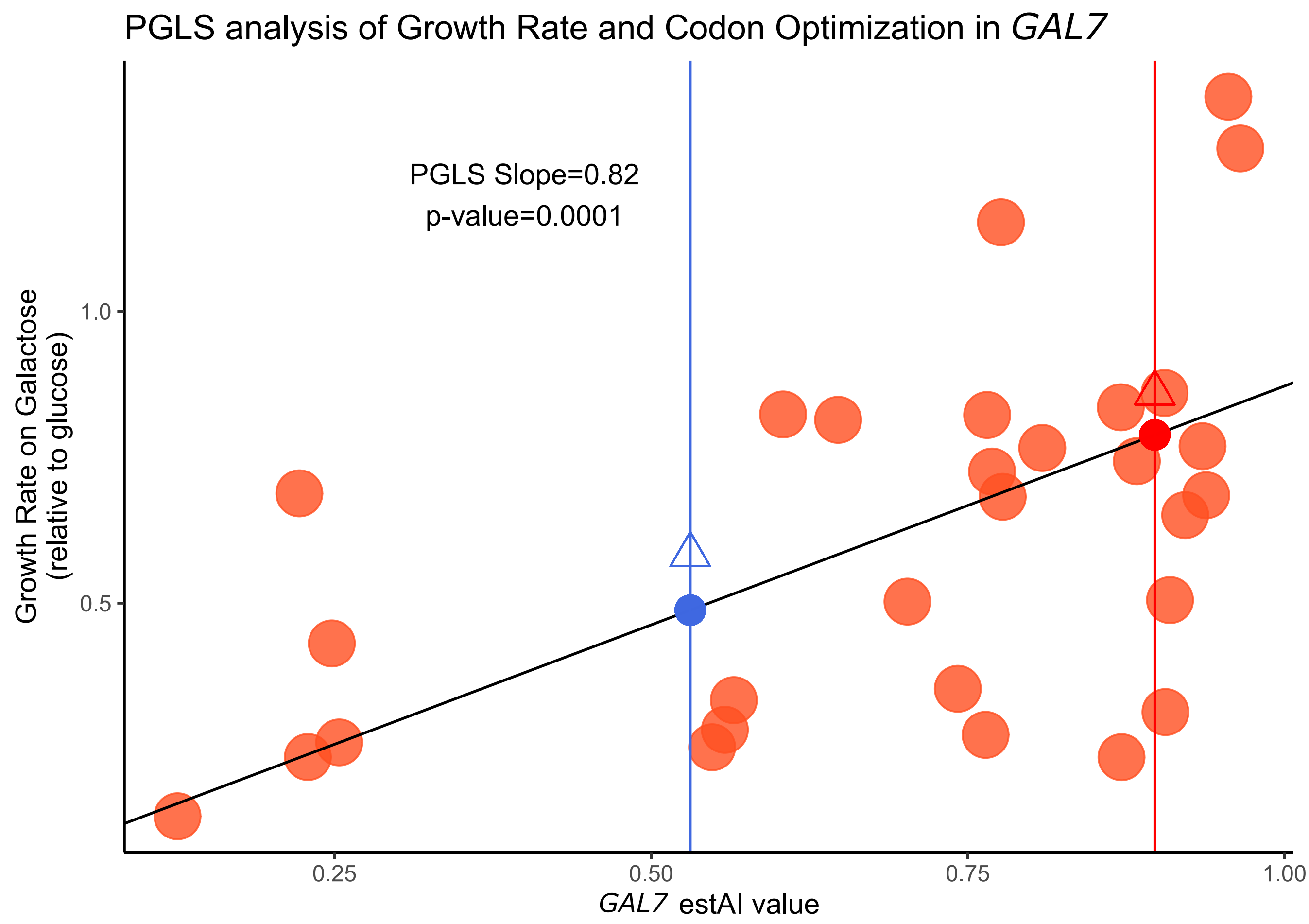
PGLS analysis of Growth Rate and Codon Optimization in GAL7
PGLS Slope=0.82
p-value=0.0001
Growth Rate on Galactose
(relative to glucose)
1.0
0.5
0.25
0.50
0.75
1.00
GAL7 estAI value

C

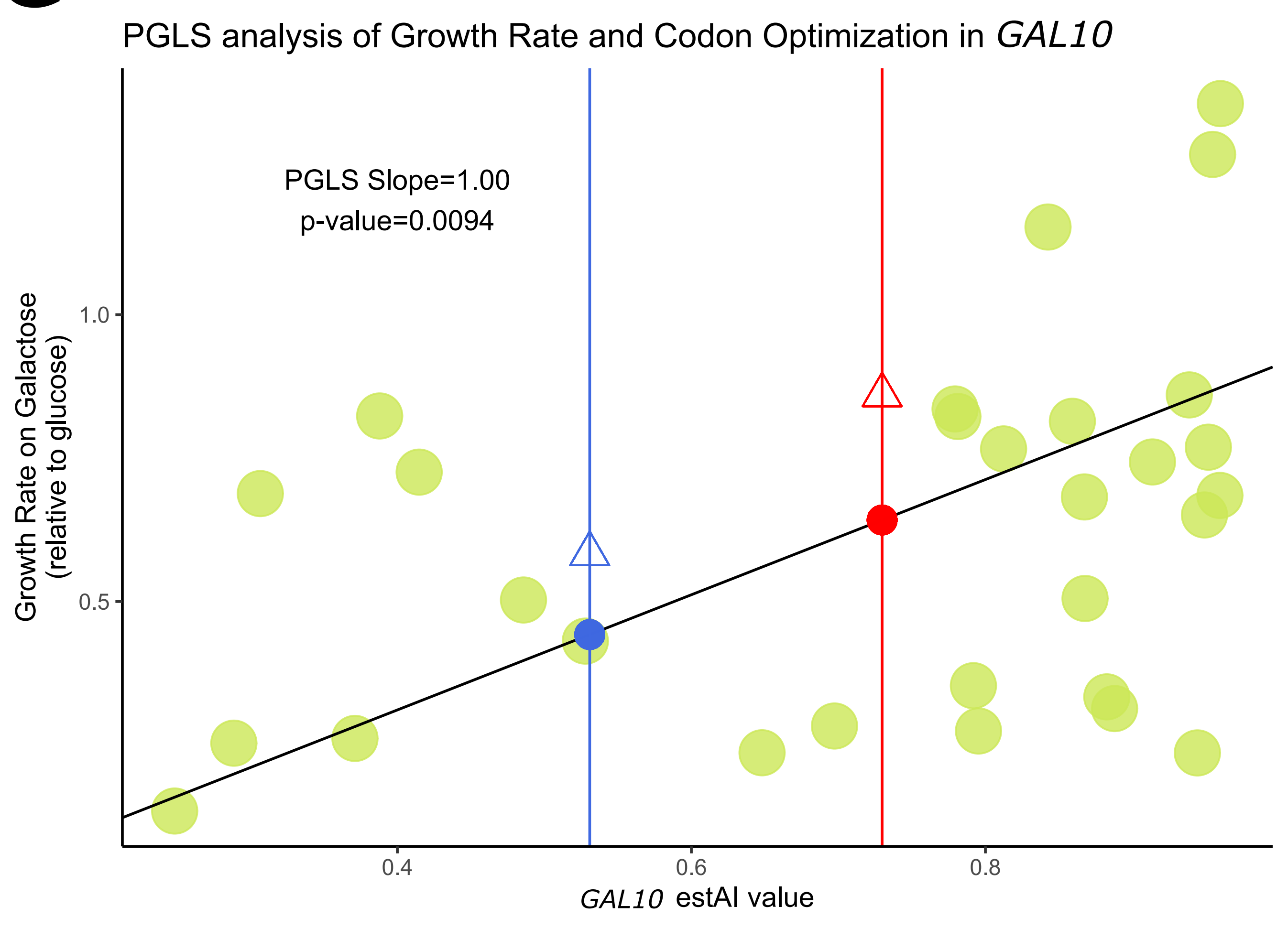
PGLS analysis of Growth Rate and Codon Optimization in GAL10
PGLS Slope=1.00
p-value=0.0094
Growth Rate on Galactose
(relative to glucose)
1.0
0.5
0.4
0.6
0.8
GAL10 estAI value
