# Supplementary material for: Signatures of optimal codon usage in metabolic genes inform budding yeast ecology
Source: PLoS Biol. 2021 Apr 19;19(4):e3001185. doi: 10.1371/journal.pbio.3001185 (PMC8084343; doi:10.1371/journal.pbio.3001185)
Supplement: Attachment — Submitted filename: GAL_codon_Response_FINAL.pdf [file pbio.3001185.s017.pdf]

Academic Editor:

Like the reviewers, I found the paper very interesting, but there is a bit of overselling of the implications. I recommend a major revision. Some of the recommendations by reviewers 3 and 4 are unrealistic as they would require extensive experimental work that would substantially delay any potential publication. Below are my reactions to reviewer comment and suggestions :

We thank the editor for distilling down the reviewers' key points and providing us with guidance on how to handle them. We have found this distillation incredibly helpful and have addressed each individual point below.

Rev #3: "Is GAL gene optimization not correlated with growth under other (non-galactose) growth conditions?"

This comment highlights that lack of certain control experiments. It is important to demonstrate that codon adaptation in GAL gene is specific to galactose growth. This requires measuring growth rate in other media in the laboratory.

Great question. We have conducted this control experiment and found that *GAL* gene optimization is not correlated with growth in another condition. Specifically, we compared codon optimization in the *GAL* genes with growth rate on medium containing mannose (after adjusting for phylogenetic relatedness) and found that there was no significant correlation (p-values of 0.975, 0.920, and 0.112 in *GAL1*, *GAL10*, and *GAL7*, respectively)**.** These new results are now included in the results section of the manuscript on lines 440 to 454.

Another important and feasible test is to demonstrate that codon adaptation of metabolic genes (not directly involved in galactose utilization) does not correlate with growth rate in galactose medium. This would require bioinformatic analysis only. This test is all the more important as certain species may be better able to optimize codon usage in all highly expressed genes due to differences in population size, regardless of the specific ecological conditions they have adapted to during evolution.

Another great question. We have conducted this bioinformatic analysis and found that codon optimization of the gene encoding the key enzyme in mannose metabolism, *PMI40*, is not correlated with growth rate on galactose congaing medium (p-value of 0.606). These new results are now included in the results section of the manuscript on lines 440 to 454

Rev #3: "In S. cerevisiae, or other species in which this may have been explored, does increased GAL gene optimality (or increased genes expression) lead to better growth on galactose? I appreciate that insufficient expression would slow growth, but is more necessarily better? I suspect there is a point of diminishing returns or a point where 'enough' gene product is made for optimum growth. Does optimality drive the genes above that point? Would the genes still be there without the optimality?"

This is a lot of work, as it demands generating mutant strain with altered codon usage of the GAL genes. However, this highlights that the main conclusions on the **predictive power** of the method should be toned down.

We have addressed this comment in three different ways. First, we have elaborated on the literature on galactose induction in budding yeasts. Most notably, the closely related species *Saccharomyces cerevisiae* and *Saccharomyces uvarum* exhibit different growth phenotypes in galactose: *S. uvarum* grows more robustly in galactose than *S. cerevisiae* (https://pubmed.ncbi.nlm.nih.gov/23852385/, https://pubmed.ncbi.nlm.nih.gov/26863195/, https://pubmed.ncbi.nlm.nih.gov/27690225/, https://pubmed.ncbi.nlm.nih.gov/29788479/). While the *GAL* genes of *S. cerevisiae* and *S. uvarum* show high amino acid sequence identity (88.6%, 87.4%, and 88.6% for *GAL1*, *GAL10*, and *GAL7*, respectively), all three are more optimized in *S. uvarum* than in *S. cerevisiae* (0.78 vs. 0.70 for *GAL1*, 0.66 vs. 0.46 for *GAL10*, and 0.55 vs. 0.33 for *GAL10*). These results, which are now discussed on lines 406-416 in the section titled "GAL codon optimization is correlated with growth rate on galactose" of the revised manuscript, provide additional support for the association between current codon optimization values and growth on galactose.

Second, we agree with the reviewer that there is likely a point at which there would be diminishing returns on the optimizing of expression of the *GAL* genes, especially as metabolic intermediates accumulate and the glycolytic load within the cell increases. The precise mechanistic role of codon optimization in the process of galactose metabolism is likely more complicated than simply improving the translational efficiency of the mRNA transcript (for a review of the diverse roles of codons in protein production, see https://pubmed.ncbi.nlm.nih.gov/33010203/). However, we generally expect that codon optimization will remove codon usage as a barrier to reaching the maximum flux possible through this pathway. We have now discussed this point on lines 406-416 in the section titled "GAL codon optimization is correlated with growth rate on galactose" of the revised manuscript.

Finally, we have clarified that codon optimization can be viewed both as a contributing factor in gene expression (mechanistic role) and as an adaptation (evolutionary role). Briefly, the correlation between gene expression and codon optimization results from selection acting on codon usage (most likely, this selection is sufficiently strong to alter codon usage only in genes that have been moderately or highly expressed over a species' evolutionary history). Therefore, this correlation stems from not just *how highly* expressed the gene is when the organism is grown on galactose, but also from *how well adapted* the organism is for galactose utilization. Organisms that are better adapted for galactose utilization will experience higher selective pressure on codon optimization in the *GAL* pathway. Therefore, **the correlation we observe between high codon optimization and growth rate on galactose is likely reflective of both the mechanistic and evolutionary roles of codon usage**. Species with an evolutionary history of growth on galactose will likely grow faster on this substrate and have experienced an adaptation at the level of codon optimization. This is now featured in **Figure 6** and in the "Conclusion section" on lines 682-687 of the revised manuscript.

I agree with reviewer 4 that “the presented results do not strictly assess how ecological variables can be predicted from the genomic pattern of codon usage. Such an approach would require at least some kind of **linear modelling allowing to quantify the prediction error and some cross-validation approach** to assess how accurate such prediction would be. “ Reviewer 2 also highlighted this problem: “But these hypotheses and predictions are actually not tested experimentally, leaving the validity of the approach (and of the initial hypothesis) not fully convincing.”

We agree with you and the reviewers and think that the important control experiments they suggested offer evidence that codon optimization is specific to the metabolic pathway and phenotype of interest. We have added a section in the results – “Can we predict growth rate on galactose from GAL codon optimization data?” from lines 457 to 475

In addition to the control experiments described above, we have added a predictive analysis of two new organisms, *Kluyveromyces wickerhamii* (Saccharomycetaceae) and *Wickerhamiella occidentalis* (Dipodascaceae/Trichomonascaceae clade). By characterizing the codon optimization of the *GAL*actose metabolism genes of the two species’ genomes, we used a phylogenetically-corrected regression analysis inferred on the data of all previously characterized species to predict each species’ growth rate on galactose. To experimentally test our predictions, we measured quantitative growth rate in galactose media of both strains in the laboratory. In *K. wickerhamii*, we predicted growth rates of 0.65, 0.47, and 0.44 based on *GAL1*, *GAL7*, and *GAL10* sequences, respectively. The growth rate measured in the laboratory was 0.58. In *W. occidentalis*, we predicted growth rates of 0.53, 0.76, and 0.54 based on *GAL1*, *GAL7*, and *GAL10* sequences, respectively. The growth rate of *W. occidentalis* measured in the laboratory was 0.86.

To better understand the performance of our growth rate predictions based on codon usage, we tested how well *GAL*actose genes with randomly assigned codons could predict growth rate. For each *GAL* gene associated with *K. wickerhamii* and *W. occidentalis*, we generated 1,000 DNA sequences with random codon usage (while keeping the protein sequence identical). For each random sample, we then predicted growth rate based on our regression analysis. In both cases, both the predicted and the actual growth rates fall outside of the 95th percentile (see figure below). Additionally, for all but one observation (*GAL7* in *K. wickerhamii*) the predictions and actual growth rate fall outside the 99th percentile. These results show that our predictions are highly informative.

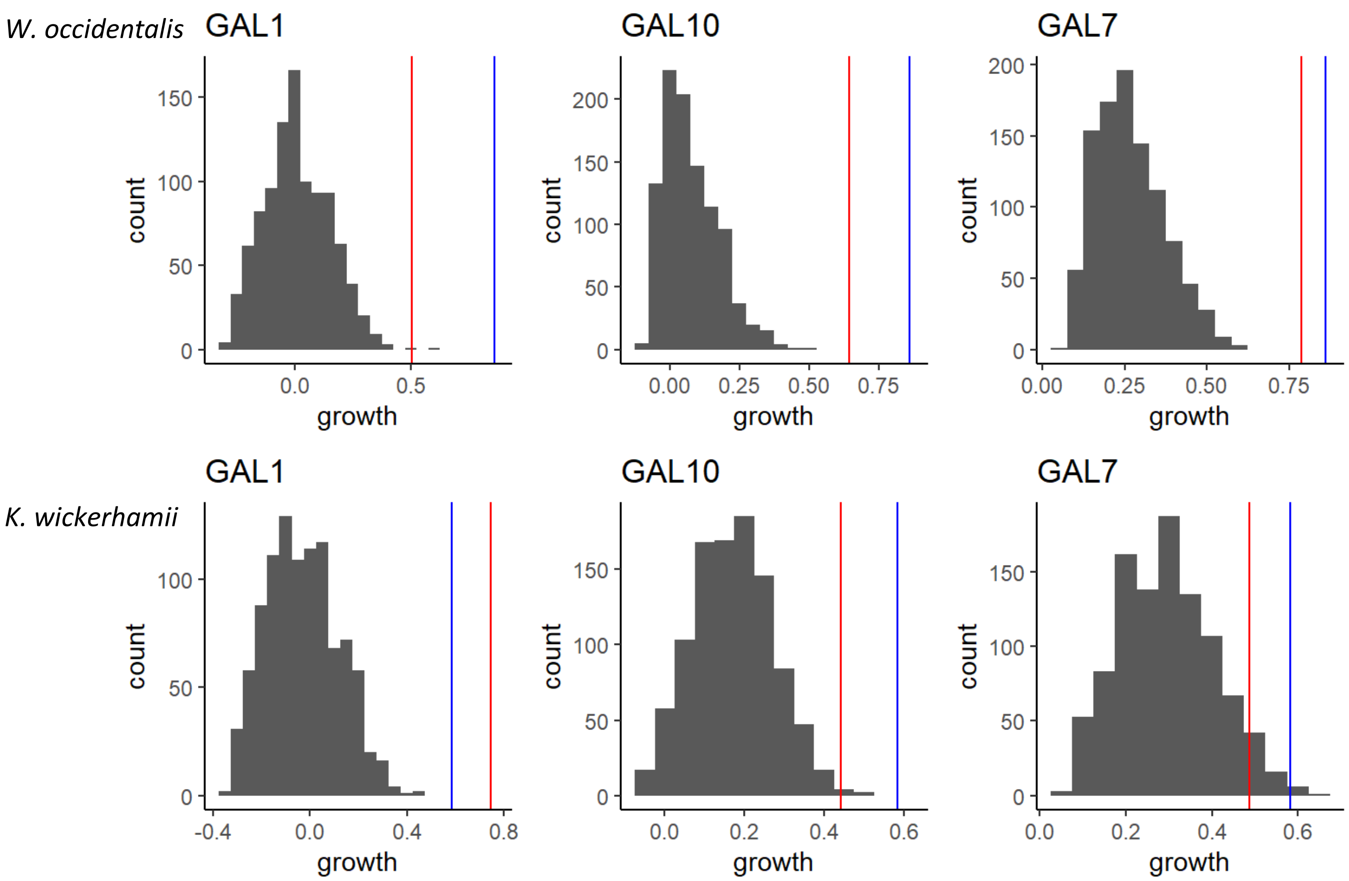

Figure: Distribution of predicted growth rates based on random codon assignment in the GAL*actose genes. Red line: predicted growth rate based on actual codon composition. Blue line: empirically measured growth rate. In all analyses, both the predicted and the actual growth rates fall outside of the 95th percentile of the random codon assignment distribution.*

While neither estimate is perfect, these results suggest that the simple phylogenetic linear model we constructed based on codon optimization of a pathway's gene has predictive power for growth phenotypes. It is likely that a more sophisticated model using more genomes and multiple genes within the major clades would provide even more accurate estimates. Furthermore, *K. wickerhamii* belongs to the Saccharomycetaceae clade in which we have made ecological predictions. The optimization of *K. wickerhamii GAL* genes lie below the lower quantile of species found in dairy-associated niches: *GAL1* below 0.892 (observed 0.887), *GAL10* below 0.843 (observed 0.660), and *GAL7* below 0.776 (observed 0.531). We would therefore predict that *K. wickerhamii* is not associated with dairy niches. Indeed, examination of the literature suggests that *K. wickerhamii* is "confined to *Drosophila* species and to tree exudates that probably act as *Drosophila* habitats" (1. Kurtzman, C.P., Fell JW. The yeasts a taxonomic study 5th edn. Elsevier Science Publishers, Amsterdam. The Yeasts. 2011.)

These results and figure are now featured in section "Can we predict growth rate on galactose from GAL codon optimization data?" from lines 457 to 475 of the revised manuscript.

Therefore, the claims below should be toned down:
“This work highlights the potential of codon optimization as a tool for gaining insights into the metabolic ecology of microbial eukaryotes. Doing so may be especially illuminating for studying fungal dark matter—species that have yet to be cultured in the lab or have only been identified by genomic material. “

We agree with the reviewers and editor that additional experimental and predictive analyses are necessary to support this statement. We have revised and toned down this sentence (lines 46-49).

Reviewer #1: Signatures of optimal codon usage predicts metabolic ecology in budding yeasts.

The submission from La Bella describes a detailed analysis of the association with optimal codon usage in the galactose metabolism pathway and ecological niche for 329 budding yeast species. The study exploits the diversity of whole genome sequences available for yeast species, many of which were originally obtaining by the authors' labs, and their earlier estimations of codon usage bias from 2019. There have been many studies of the evolution of the galactose gene cluster in yeasts, but this is one of the first (and certainly the most extensive) studies of codon adaptation, as a proxy for gene expression. The authors find that species with optimal codon usage in GAL genes (high expression) are associated with dairy environments (Saccharomycetaceae) or with humans (CUG-Ser1 clade). They also find a correlation between codon optimization in GAL genes and in thiamine biosynthesis genes, suggesting that there is a biochemical or ecological connection. The study is clear, well described and original, though the **ability to predict metabolic ecology is a little overstated**. For the CUG-Ser1 clade for example, it looks like it might be difficult to predict if newly identified species are associated with humans. The analysis is likely to be of general interest, including to yeast biologists, and metabolic ecologists.

We thank the reviewer for their comments. We agree that we overstated our predictive ability and have toned down our claims throughout the revised manuscript. We also agree that a single pathway is unlikely to give us the power to predict ecology. Our ongoing work aims to use the codon optimization of genes to make testable predictions on organisms’ metabolic ecology.

Specific points.

1. Figure 1 is central to the analysis, but it is impossible to read in the printed version. Zooming in on the PDF does reveal the unfilled symbols, but they are impossible to see in a printout. It is also very difficult to see the variation in estA1 measurements using the color scale, and it is impossible to identify specific species without reference to earlier papers from the group. For example, I cannot easily pick out the human-enriched species with optimal codon usage referred to from lines 389. Some additional panels, or supplementary figures, would help (similar to Fig. 4). Fig 1B is too small relative to 1A.

We apologize for the lack of resolution of the figure. To improve the figure's resolution, we have removed species from Figure 1 that do not metabolize galactose. This reduced the number of species and has made all aspects of figure 1 easier to read. The full tree, which has been modified to show the human-enriched species, has been moved to our supplemental materials (Supplementary Figure 1).

2. I have some problems understanding the correlation with the thiamine biosynthesis pathway, or more accurately, in understanding why K14154 was selected for discussion. As the authors note, this is number 8 in the list (Supplementary Table 3). Number 3 (K02564) is NAG1, a glucosamine-6-phosphate deaminase, involved in sugar (glucosamine) metabolism. Is it not more likely that correlation of galactose and glucosamine metabolism may be more important for ecology? NAG genes also tend to be clustered. Perhaps the authors have already examined codon optimization in NAG genes, which is worth a comment. Glucosamine metabolism may also be associated with first gene in the list (glycan synthesis). Number four, K01077 is PHO8 which also has role in thiamine synthesis, and could be added to Fig 5A. It would be useful to include KO definitions in Supplementary table 3.

We thank the reviewer for their insights into the *NAG* and *PHO8* genes. Given the interest in many of the other pathways identified in the KEGG analysis, we have broadened the scope of our reporting to include discussion of all the top KEGG orthologs identified. We now discuss the genes *SNF3/RTG2/GAL2, GPI16, SHP1, YCK1/YCK2, ICL1, and THI6/PHO8*. This additional discussion can be found in the section "GAL optimization is correlated with optimization in other metabolic genes" from lines 621 to 665 of the revised manuscript. We have added the KEGG ortholog definitions to Supplementary Table 3.

Minor

3. Is the lack of GAL clusters/galactose metabolism in the Pichiaceae (spelled incorrectly in Fig. 1) and the Phaffomycetaceae discussed elsewhere?

Yes, the loss of galactose metabolism and subsequent reacquisition of the *GAL*actose pathway via horizontal gene transfer has been extensively examined in previous work including in this recently accepted publication: Haase et al. Repeated horizontal transfer of GALactose metabolism genes violates Dollo's law of irreversible loss. *Genetics*: in press. DOI of *bioRxiv* preprint: https://doi.org/10.1101/2020.07.22.216101. We have noted this novel finding in the figure legend of Figure 1**.** We have also corrected the typo in Fig. 1.

4. The ecological enrichment (Fig. 3) is certainly interesting. Are the authors suggesting that changes in optimal codon usage occurred very recently (in evolutionary terms), because dairy associations are likely to be recent?

The speed at which codon optimization occurs within a genome or gene remains unclear. The addition of the remaining 700+ known yeast species to this analysis could help clarify this question. Furthermore, codon optimization in the *GAL*actose pathway due to other unknown

evolutionary pressures could pre-adapt species to the dairy environment, allowing them to invade this habitat as it became available.

5. Page numbers are missing from references.

We have corrected the reference style to include page numbers or DOI.

Reviewer #2: The manuscript by LaBella et al. aims at testing the hypothesis that codon optimality and gene function can be used to infer the ecology of yeasts. Such reverse ecology approaches are particularly interesting since ecological data show strong sampling -and reporting- bias. Codon optimilaty is an underused genomic signature which can be accessed in a relatively straightforward manner, given recent development in sequencing technologies. The approach is not whithout precedence: it was used very effectively, and experimentally validated, in bacteria to discover new genes involved in oxidative stress tolerance (doi: 10.1186/gb-2014-15-3-r44). It was also applied, and partly validated, on metagenomic data (https://pubmed.ncbi.nlm.nih.gov/23921637/) and used recently on ecosystem data (doi: 10.7554/eLife.49816). Extending the approach to eukaryotic microbes is undoubtely promising. Unfortunately, the study in its current state compares somewhat unfavorably to the works listed above in some aspects. Starting from the general hypothesis that signatures of codon optimality carry information on yeasts ecology, the authors provide a generally rigorous and well conducted analysis of codon optimality in GAL genes from the whole subphylum of budding yeasts. I very much appreciated that the authors used phylogenetic independent contrast to avoid lineage-dependent bias in their estimates of codon optimality. These analyses allow the authors to generate new, often experimentally tractable predictions (adaptation of Naumovozyma castellii and Kazachstania unispora to dairy environment, the association of thiamine metabolism with growth on galactose or in diary -but not alcohol- environment). But these hypotheses and predictions **are actually not tested experimentally**, leaving the validity of the approach (and of the initial hypothesis) not fully convincing. For this major reason, I feel like the current manuscript is too preliminary for publication, but represents a solid basis and promising lead.

We thank the reviewer for their insight and appreciation of the utility of codon usage. We agree that this work is intended to provide a foundation for future work, including experimental validation of the role of codon usage in metabolic pathways. We have added two additional analyses to strengthen the validity of our findings. First, we have added two negative control experiments to illustrate the specificity of our optimization versus growth rate correlation. We have conducted this bioinformatic analysis and found that codon optimization of the gene encoding the key enzyme in mannose metabolism, PMI40, is not correlated with growth rate on galactose congaing medium (p-value of 0.606). These new results are now included in the results section of the manuscript on lines 440 to 454. We also compared codon optimization in the GAL genes with growth rate on medium containing mannose (after adjusting for phylogenetic relatedness) and found that there was no significant correlation (p-values of 0.975, 0.920, and

0.112 in GAL1, GAL10, and GAL7, respectively). These new results are now included in the results section of the manuscript on lines 440 to 454.

Second, we have added a predictive analysis of two new organisms, Kluyveromyces wickerhamii (Saccharomycetaceae) and Wickerhamiella occidentalis (Dipodascaceae/Trichomonascaceae clade). By characterizing the codon optimization of the GALactose metabolism genes of the two species' genomes, we used a phylogenetically-corrected regression analysis inferred on the data of all previously characterized species to predict each species' growth rate on galactose. To experimentally test our predictions, we measured quantitative growth rate in galactose media of both strains in the laboratory. In K. wickerhamii, we predicted growth rates of 0.65, 0.47, and 0.44 based on GAL1, GAL7, and GAL10 sequences, respectively. The growth rate measured in the laboratory was 0.58. In W. occidentalis, we predicted growth rates of 0.53, 0.76, and 0.54 based on GAL1, GAL7, and GAL10 sequences, respectively. The growth rate of W. occidentalis measured in the laboratory was 0.86.

To better understand the performance of our growth rate predictions based on codon usage, we tested how well GALactose genes with randomly assigned codons could predict growth rate. For each GAL gene associated with K. wickerhamii and W. occidentalis, we generated 1,000 DNA sequences with random codon usage (while keeping the protein sequence identical). For each random sample, we then predicted growth rate based on our regression analysis. In both cases, both the predicted and the actual growth rates fall outside of the 95th percentile (see figure below). Additionally, for all but one observation (GAL7 in K. wickerhamii) the predictions and actual growth rate fall outside the 99th percentile. These results show that our predictions are highly informative.

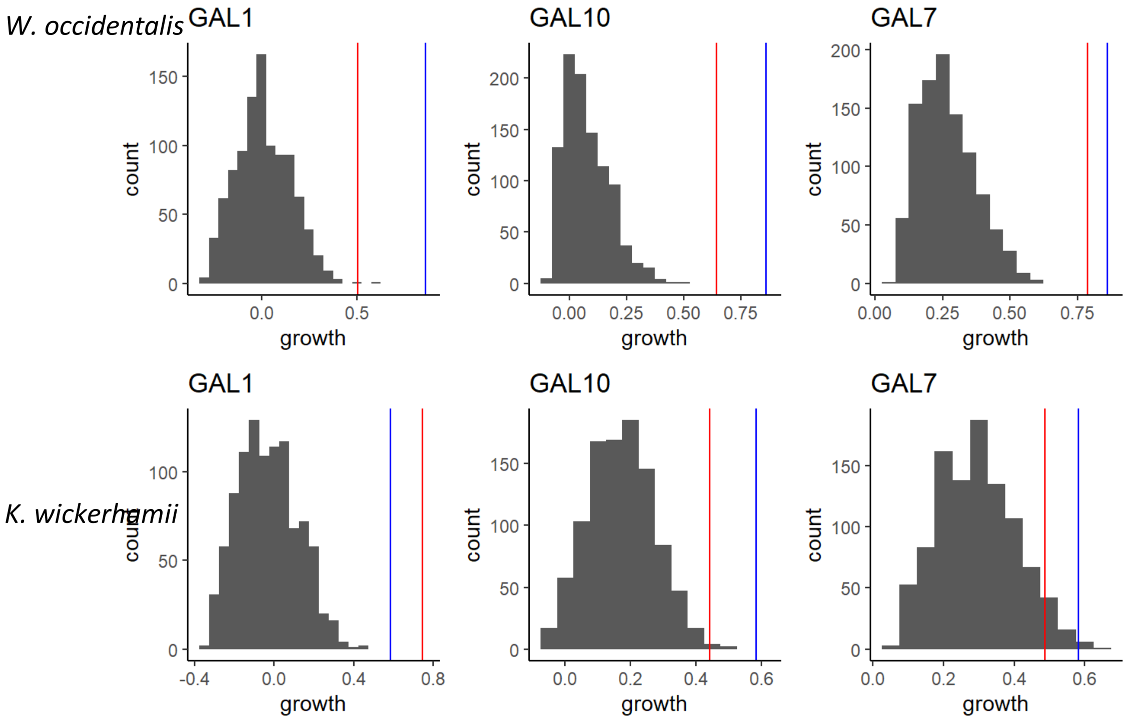

Figure: Distribution of predicted growth rates based on random codon assignment in the GALactose genes. Red line: predicted growth rate based on actual codon composition. Blue line: empirically measured growth rate. In all analyses, both the predicted and the actual growth rates fall outside of the 95th percentile of the random codon assignment distribution.

While neither estimate is perfect, these results suggest that the simple phylogenetic linear model we constructed based on codon optimization of a pathway's gene has predictive power for growth phenotypes. It is likely that a more sophisticated model using more genomes and multiple genes within the major clades would provide even more accurate estimates. Furthermore, K. wickerhamii belongs to the Saccharomycetaceae clade in which we have made ecological predictions. The optimization of K. wickerhamii GAL genes lie below the lower quantile of species found in dairy-associated niches: GAL1 below 0.892 (observed 0.887), GAL10 below 0.843 (observed 0.660), and GAL7 below 0.776 (observed 0.531). We would therefore predict that K. wickerhamii is not associated with dairy niches. Indeed, examination of the literature suggests that K. wickerhamii is "confined to Drosophila species and to tree exudates that probably act as Drosophila habitats" (1. Kurtzman, C.P., Fell JW. The yeasts a taxonomic study 5th edn. Elsevier Science Publishers, Amsterdam. The Yeasts. 2011.)

These results and figure are now featured in section "Can we predict growth rate on galactose from GAL codon optimization data?" from lines 457 to 475 of the revised manuscript.

Major concerns:

RESULTS

1. The predictions generated by the analysis presented here are not validated experimentally. Therefore, there is no evidence to support the validity of the reverse ecology approach proposed. All predictions are probably not experimentally tractable, however some of the following should be tested:
. Deoptimize GAL genes or thiamine biosynthesis genes and check if there is a phenotypic effect specifically in the identified niche.
. Test for the expression of GAL genes or accumulation of GAL proteins in some of the species and environment of interest . L420 suggests that Spathaspora species would be "enable[d] or predispose[d them] to colonize human hosts". Any experiment to get support for this hypothesis?
. L452 "It is likely that [species] associated with both dairy and alcohol niches contain[s] species or populations that are better adapted to one niche than the other". Can't this be tested experimentally to validate the hypothesis?

We agree with the reviewer that all of these proposed experiments are interesting, particularly transgenic optimization/deoptimization of the pathway. However, as suggested by the editor, we believe that these experiments are outside the scope of this manuscript. To provide additional support for the validity of our approach, we measured additional growth rate traits as negative controls and conducted computational predictions of growth and ecology.

2. "GAL codon optimization is correlated with growth rate on galactose". Correlation is not very convincing as it seems strongly driven by extreme values, especially for GAL7 where 2 species with low PIC are probably responsible for most of the correlation. In agreement, when CUG-Ser1 and sccharomycotina clades are considered separately, only GAL7 correlation remains significant in both clades. And even there, three species are probably strong drivers in the correlation observed for the two clades. The same concern applies to data presented in Figure 5B.

We appreciate the reviewer's feedback and have clarified the interpretation of the phylogenetically independent contrasts in the legend of Figure 2. We have tested for the influence of outlier species values using a residual analysis, which led to the removal of *Metschnikowia matae* var. *matae*. The points on the PIC graph represent the comparison between two phylogenetically independent contrasts (including nodes and leaves) for each metric, and not species themselves. If you consider a tree A(B,C) where node X connects B and C, there would be two phylogenetically independent contrasts B versus C (contrast 1) and X versus A (contrast 2). Therefore, the "extreme" or "outlier" points on the plot represent coevolution of the two variables to more extreme values.

To test whether these correlations are specific to galactose, we have added two negative controls: comparison of growth rate on mannose-containing medium versus *GAL* codon optimization, and comparison of mannose metabolism gene optimization versus growth rate on galactose. We found that codon optimization of the gene encoding the key enzyme in mannose metabolism, PMI40, is not correlated with growth rate on galactose congaing medium (p-value of 0.606). These new results are now included in the results section of the manuscript on lines 440 to 454. We also compared codon optimization in the GAL genes with growth rate on medium containing mannose (after adjusting for phylogenetic relatedness) and found that there was no significant correlation (p-values of 0.975, 0.920, and 0.112 in GAL1, GAL10, and GAL7, respectively). These new results are now included in the results section of the manuscript on lines 440 to 454.

3. "GAL codon optimization is associated with specific ecological niches". The authors conclude that GAL genes optimization is higher in species from human-associated niches. Yet, codon optimization in species from human and/or insect niche is not significantly higher than that of species from neither human or insect niche. An alternative interpretation could be that association with insect decreases codon optimization. Can the authors exclude this alternative?

There are several hypotheses for the differences in codon optimization observed between environments: 1) there is selection for increased optimization in *GAL*alctose genes in human-associated yeasts, 2) there is relaxation in selection on optimization in *GAL*actose in insect-only associated yeasts, or 3) There is selection on de-optimization in the *GAL*actose genes in insect-only associated yeasts.

In all of these scenarios selection on codon usage is associated with niche specificity. To distinguish between these scenarios, we would need to know both the ancestral niches and optimization states—which we cannot accurately estimate with our current sample. Regardless of the evolutionary direction, codon usage of *GAL* genes in human/insect- and dairy-associated niches is optimized *compared to the rest of the genes in each individual species' genome*. This is evidenced by the high (~0.75 or greater) estAI values of *GAL* genes in specific environments. This suggests that *GAL* codon usage is more optimized in the genomes of species associated with humans/insects both compared to of species associated with other environments (insects only), as well as compared to the rest of the genes in each individual species' genome. Additionally, the reviewer is correct that the other (neither human nor insect) environments also appear to have codon optimization in the *GAL*actose pathway – this result however does not reveal any niche-specific information as there are only 16 species in this category. This issue has now been addressed in the section "GAL codon optimization is associated with specific ecological niches" on lines 487 to 492 of the revised manuscript.

4. Have authors noted any relatioship between codon nitrogen content and ecological niche, as shown in doi: 10.1186/s13059-016-1087-9 ?

We would like to thank the reviewer for pointing out this interesting hypothesis. Given the finding in this manuscript that "Selection acting on nitrogen content is independent of selection acting on translational efficiency", we believe it should not have a substantial impact on our results. Additionally, because our analyses are normalized within each genome to the genome-specific tRNA, GC, and codon landscapes, we would not be able to detect differences in codon usage between niches.

DISCUSSION

4. L566 "These results are especially promising as this method can be applied directly to genomic data—which is the only source of information for microbial dark matter known only from DNA". I feel like application may not be that straightforward as estimation of codon optimality often requires genome scale information, such as tRNA complement and codon adaptation values for all genes. Also ecological information can be deduced if optimilaty is high in genes with known function, which may not be very frequent. Can the authors discuss these possible limitations and how to circumvent them?

The reviewer is correct, that codon optimization calculations require genome-scale data. However, recent advances in single-cell and metagenomic sequencing have enabled the sequencing of genomes from microbial dark matter.

The reviewer is also correct that knowledge of gene function is critical for our interpretation of codon optimization analysis. We may, however, be able to identify genes with a similar general function (e.g., hydrolase or sugar transporter) and codon optimization regime within our data through sequence homology searches. We would subsequently have to functionally validate

these predictions. While we agree that these analyses might only be a start toward understanding the metabolic ecology of a microbe, our results do suggest that microbial metabolic ecology is optimized toward pathways whose functions are currently unknown, which may be very interesting from a bioprospecting perspective.

We have expanded our discussions in the Conclusion on lines 701 to 207 to make these points clear.

5. Can the authors discuss added value from their study in the context of previous codon optimality-based reverse ecology studies (eg doi: 10.1186/gb-2014-15-3-r44; https://nam04.safelinks.protection.outlook.com/?url=https%3A%2F%2Fdoi.org%2F10.1093%2Fnar%2Fgkt673&data=02%7C01%7Cabigail.l.labella%40vanderbilt.edu%7C41491df8d71c499a00af08d86646b2a8%7Cba5a7f39e3be4ab3b45067fa80faecad%7C0%7C0%7C637371801134321847&sdata=GgC8LpH9CUiIvG0g3CBOSxHELoX5Tp6TSLCpysKwt3o%3D&reserved=0; doi: 10.7554/eLife.49816)?

We have expanded on the discussion on the context of our study in the “Conclusions” section on lines 708-715**.** Briefly, codon optimization has been used in a reverse ecology context in multiple microbial systems. This previous work has identified general pathway categories associated with ecological adaptation but lacks specific examples of genotype-phenotype-ecology connections. Here, by focusing on one well known and extensively characterized pathway, we are able to show that codon optimization is associated with both a specific phenotype (growth rate on galactose-containing medium) and ecologies (dairy-associated and human-associated niches).

Minor comments
The manuscript is very well written and very pleasant to read. Figures are of high quality.
L498 "pathways associated with galactose optimization" > "galactose metabolism optimization"

This has been corrected.

Reviewer #3: The authors explore the use of codon optimality as a tool for reverse ecology. The authors report positive correlations between GAL gene codon optimality and growth on galactose media. The authors show that yeasts that live in an environment with lactose have higher optimality of GAL genes. The authors also found a correlation between codon optimality in GAL and thiamine biosynthesis genes and then show that thiamine genes are also especially optimized in environments with lactose.

Using codon optimality as a tool for reverse ecology is a very cool idea. It was, however, not clear to me from the paper how useful the tool is relative to other reverse ecology tools like gene presence or conservation. It would be helpful if the authors could highlight what discoveries this method allowed in this dataset, that would not have been found with other approaches.

We thank the reviewer for this comment. We have added more information about other reverse ecology tools in the Introduction on lines 87-89 and in the**.** As for gene presence/absence, we have added in information about the frequency of *GAL* gene presence/absence for the pathways of interest. We find for the Saccharomycetaceae that gene presence/absence of the *GAL* pathway is nearly identical between the three states. Of the species found in dairy, alcohol, or both environments the percent of species with a complete *GAL* pathway is 83%, 88%, and 90% for the niches, respectively. The four dairy-associated species lacking a complete *GAL* pathway (the 17%) may be utilizing the other sugars and fermentation products available in dairy (often produced by lactic acid bacteria). Therefore, whereas gene presence/absence analyses may be better suited to address whether organisms *can* grow on a given substrate, codon optimality analyses are better suited to address *how well* organisms grow on a given substrate. We have made this point in Results on lines 557-565.

I was also confused about what species were included in the correlation analyses and how the growth rates were calculated. This confusion prevented me from getting too excited about the correlations presented, especially given the low strength of the correlations. For example, for the GAL growth/GAL optimal codon correlation, I couldn't shake the idea that the correlations could be driven by **genes that were decaying** and thus had low optimality and were also not able to foster growth on galactose media. I have listed questions/comments that arose as I read to make it clear areas where I required more information/explanation to fully understand.

We have addressed these issues as they arise in the comments below.

Questions/suggestions:
In the abstract, I was confused by this sentence:
"For example, optimal codon usage of GAL genes is greater than 85% of all genes in the major human pathogen Candida albicans (CUG-Ser1 clade) and greater than 75% of genes in the dairy yeast Kluyveromyces lactis (family Saccharomycetaceae)."
-I don't know what 85% of all genes means. Is it that the GAL genes use more optimal codons than 85% of all genes? I originally read it as 85% of codons are optimized.

The stAI values (aka codon optimization values) for each gene are calculated based on the geometric mean of the tRNA adaptation index of all the codons within the gene. The codon optimization values (stAI) are reported as compared to the empirical cumulative distribution (estAI). So, a gene with an estAI value of 0.85 has an optimization value that is greater than 85% of the other genes in the genome. This has been further clarified on lines 202-209.

Is GAL gene optimization not correlated with growth under other (non-galactose) growth conditions?

We have added two additional analyses to strengthen the validity of our findings. First, we have added two negative control experiments to illustrate the specificity of our optimization versus growth rate correlation. We have conducted this bioinformatic analysis and found that codon

optimization of the gene encoding the key enzyme in mannose metabolism, PMI40, is not correlated with growth rate on galactose congaing medium (p-value of 0.606). These new results are now included in the results section of the manuscript on lines 440 to 454. We also compared codon optimization in the GAL genes with growth rate on medium containing mannose (after adjusting for phylogenetic relatedness) and found that there was no significant correlation (p-values of 0.975, 0.920, and 0.112 in GAL1, GAL10, and GAL7, respectively). These new results are now included in the results section of the manuscript on lines 440 to 454.

In the methods or elsewhere, I looked for an explanation of how codon optimality was determined for the different species. I found this:
"Codon optimization of individual GAL genes was assessed by calculating the species-specific tRNA adaptation index (stAI) from previously calculated species-specific codon relative adaptiveness (wi) value." This needs more explanation. Were there any empirical measurements that went into these values? If not, that should be stated explicitly and justified.

The procedures and analysis of codon usage throughout the entire subphylum have been previously reported in LaBella et al 2019. To address the reviewer's concern, we have added more information on the methods used to calculate codon optimization here on lines 202-209**.**

In S. cerevisiae, or other species in which this may have been explored, does increased GAL gene optimality (or increased genes expression) lead to better growth on galactose? I appreciate that insufficient expression would slow growth, but is more necessarily better? I suspect there is a point of diminishing returns or a point where 'enough' gene product is made for optimum growth. Does optimality drive the genes above that point? Would the genes still be there without the optimality?

We have addressed this comment in three different ways. First, we have elaborated on the literature on galactose induction in budding yeasts. Most notably, the closely related species Saccharomyces cerevisiae and Saccharomyces uvarum exhibit different growth phenotypes in galactose: S. uvarum grows more robustly in galactose than S. cerevisiae (https://pubmed.ncbi.nlm.nih.gov/23852385/, https://pubmed.ncbi.nlm.nih.gov/26863195/, https://pubmed.ncbi.nlm.nih.gov/27690225/, https://pubmed.ncbi.nlm.nih.gov/29788479/). While the GAL genes of S. cerevisiae and S. uvarum show high amino acid sequence identity (88.6%, 87.4%, and 88.6% for GAL1, GAL10, and GAL7, respectively), all three are more optimized in S. uvarum than in S. cerevisiae (0.78 vs. 0.70 for GAL1, 0.66 vs. 0.46 for GAL10, and 0.55 vs. 0.33 for GAL10). These results, which are now discussed on lines 406-416 in the section titled "GAL codon optimization is correlated with growth rate on galactose" of the revised manuscript, provide additional support for the association between current codon optimization values and growth on galactose.

Second, we agree with the reviewer that there is likely a point at which there would be diminishing returns on the optimizing of expression of the GAL genes, especially as metabolic intermediates accumulate and the glycolytic load within the cell increases. The precise

mechanistic role of codon optimization in the process of galactose metabolism is likely more complicated than simply improving the translational efficiency of the mRNA transcript (for a review of the diverse roles of codons in protein production, see https://pubmed.ncbi.nlm.nih.gov/33010203/). However, we generally expect that codon optimization will remove codon usage as a barrier to reaching the maximum flux possible through this pathway. We have now discussed this point on lines 406-416 in the section titled “GAL codon optimization is correlated with growth rate on galactose” of the revised manuscript.

Finally, we have clarified that codon optimization can be viewed both as a contributing factor in gene expression (mechanistic role) and as an adaptation (evolutionary role). Briefly, the correlation between gene expression and codon optimization results from selection acting on codon usage (most likely, this selection is sufficiently strong to alter codon usage only in genes that have been moderately or highly expressed over a species’ evolutionary history). Therefore, this correlation stems from not just how highly expressed the gene is when the organism is grown on galactose, but also from how well adapted the organism is for galactose utilization. Organisms that are better adapted for galactose utilization will experience higher selective pressure on codon optimization in the GAL pathway. Therefore, the correlation we observe between high codon optimization and growth rate on galactose is likely reflective of both the mechanistic and evolutionary roles of codon usage. Species with an evolutionary history of growth on galactose will likely grow faster on this substrate and have experienced an adaptation at the level of codon optimization. This is now featured in Figure 6 and in the “Conclusion section” on lines 682-687 of the revised manuscript.

"A total of 49 species' genomes had multiple copies of at least one GAL gene." Did that correlate with growth on Galactose? If not, maybe increased expression doesn't equate to optimal growth. Are the results the same without those 49?

Of the 49 species with multiple copies of *GAL* genes, the majority (30/49) belong to the clade of Saccharomycetaceae species that underwent a genome duplication event about 100 million years ago. The fate of some *GAL* genes after genome duplication has been explored in Wolfe et al 2015 (https://pubmed.ncbi.nlm.nih.gov/26066552/) and Kuang et al 2016 (https://pubmed.ncbi.nlm.nih.gov/27690225/). The vast majority, however, of *GAL* gene duplications have not been characterized functionally. The exceptions are *GAL1/3* and *GAL80/80B*, where there is considerable functional differentiation (subfunctionalization/escape from adaptive conflict in the case of *GAL1/3*; gene expression and regulatory divergence in the case of *GAL80/80B*), so we have opted to be cautious about assuming that the presence of a second copy is indicative of an increase in a gene’s activity.

In our analysis, we retained all copies of the *GAL* genes within the genome, except the *GAL1* ohnologs that have been well studied and annotated as *GAL3*, which encodes a co-inducer that often lacks enzymatic activity. We then selected the most highly optimized gene for analysis as this would likely remove ohnologs that are decaying. For example, in *Kazachstania spencerorum*, there are two copies of *GAL1:* one that is highly optimized with an estAI of .97

and one that has very low optimization with an estAI of 0.20. **If the majority of the ohnologs have diversified in function or are undergoing decay, we would expect that there would not be a significant relationship between copy number and growth rate**.

To test if growth rate on galactose containing medium is greater in species with multiple copies of the *GAL* genes, we conducted a comparative analysis using generalized estimating equations as described by Paradis and Claude (2002 https://pubmed.ncbi.nlm.nih.gov/12381290/) and conducted in R using the package ape. This test allowed us to phylogenetically compare categorial data (single versus multiple *GAL* genes) and continuous data (growth rate). Within the Saccharomycetaceae, the presence of multiple *GAL* gene copies showed no significant correlation with growth rate on galactose-containing medium. This analysis did not include the known *GAL3* sequences in six of the species studied, but included all other duplicates whose function has not been characterized. The lack of correlation between growth rate and *GAL* copy number could either be due to loss of function or divergence of function in *GAL* gene copies or a lack of correlation between expression and growth rate. These results are now presented in lines 223 to 230 in the section "Codon Optimization in the GAL pathway. "

Of the 94 species with an s-value suitable for codon optimization analysis, 20 had more than one copy of at least one *GAL* gene. We removed these 20 species and conducted the PIC analysis again. Using the 74 remaining species, the correlation between *GAL7* and growth rate remained significant (p-value of 0.0001 and slope of 0.6882272), but the correlation between growth rate and *GAL1* or *GAL10* did not, presumably because the 20 species' removal reduced our power. If instead of taking the maximum codon optimization value for species with multiple copies, we took the mean, we retained the correlation between growth rate and codon optimization in both *GAL10* and *GAL7* (p values of $4.60e^{-09}$ and 0.044). Both of these analyses are consistent with the known diversification of *GAL1* after the genome duplication. We have included the first analysis in our manuscript but have left out the second (74 species) because these new results have considerably reduced power and would require extensive discussion of the mean versus maximum that would be unnecessarily confusing.

Growth rate measurements require better explanation. "The growth rates for these species are 0.129, 0.339, and 0.211…" What do those rates mean? I am used to thinking of growth rate in terms of doubling time. How were the gal rates normalized with glucose rates?

Initial cell concentrations were not normalized to a particular optical density. Additionally, cell size can bias optical density readings. To minimize these effects, we normalized growth data to growth on glucose. This was done by first calculating the growth rate, which is represented by the maximum slope of the growth curve. These values were then normalized relative to the growth rate on glucose. Values greater than one represent growth faster than what was seen on glucose. We have expanded the methods section "Galactose Growth Data" on lines 247 to 264 and in the new Supplementary Figure 2.

"To determine if there is an association between codon optimization and the ability to grow on

galactose, we compared optimization in the GAL pathway between species that are able and unable to grow on galactose. We found that species without evidence for growth on galactose had significantly lower ($p < 0.05$) codon optimization in GAL1 and GAL7 (Supplementary Figure 4)." Is this observed if one considers only genomes that have all the genes? If not, optimality is presented as a new way to find ecological info. **Gene presence/absence is another way. I am curious if the optimality finds something gene presence/absence misses**. Or, is the low optimality signal in the things not growing on glucose being dragged down by low optimality in a decaying pathway already missing components.

We thank the reviewer for this suggestion. The analysis presented in Supplementary Figure 4 does not include species with partial *GAL* pathways. We have now included an additional analysis in Supplementary Figure 6 that compares optimization between species with complete and partial *GAL* pathways. These results show further decreases in codon optimization in species with a partial pathway.

Together these analyses suggest we may be able to better predict growth phenotypes from genomics if we incorporate codon optimization values. This low codon optimization in species with inactive pathways may be due to decay within the pathway that results in loss of codon optimization. We also note that some species only grow on galactose at specific and generally lower concentrations (https://www.ncbi.nlm.nih.gov/pmc/articles/PMC6063270/), a phenomenon whose mechanism is still under investigation. Thus, whereas gene presence/absence analyses are better suited to address whether organisms *can* grow on a given substrate, codon optimality analyses are better suited to address *how well* organisms grow on a given substrate. We have made this point in the section "GAL codon optimization is associated with specific ecological niches" on lines 557-565.

Line 355 "We found a significant positive correlation between growth rate on galactose-containing medium and codon optimization in the GAL pathway of genomes that have experienced translational selection on codon usage" Does this analysis and the others that follow exclude the species that do not grow on Gal? I know optimality is important, but low optimality seems unlikely to lead to no growth. But that no growth phenotype could be inflating the importance of optimality in the correlations. If those species were not excluded, I would like to see that analysis too.

This analysis excludes species that were not able to grow on galactose. This has been clarified in the legend of Figure 2.

Line 378: There needs to be some more up-front explanation why the discussed niches might need different GAL gene expression. There are a lot of observations that are listed in this section, but I needed more explanation as to why those differences might be important to get the most out of reading the list. The Saccharomycetaceae analyses beginning on line 437 are more self-explanatory.

We have adjusted the language to clarify that the initial examination of 50 ecological niches was not conducted to test a hypothesis for any particular association. This broad dataset allowed us to narrow down niches of interest and preform a more exhaustive literature search for niche presence/absence data.

We have clarified the subsection "CUG-Ser1 clade" within "GAL codon optimization is associated with specific ecological niches"

What were the other things that were positively correlated with GAL gene optimality and why focus on the 8th in the list?

Given the feedback from all of the reviewers, we have expanded the presentation of the KEGG data to include more of the findings. The new presentation of the results can be found in the section "GAL optimization is correlated with optimization in other metabolic genes" and in the newly added Table 1.

Reviewer #4: In this manuscript, LaBella and co-authors investigate how codon optimization (which can be computed from a complete genome sequence) can be used to predict ecological characteristics of budding yeasts. The underlying assumption is that the amount of codon usage optimization is a proxy for the level of gene expression, which in turns underlies the phenotype. More specifically, codon optimization allows pointing at highly expressed genes, and such genes are very likely to be of ecological importance. The authors illustrate this principle using three genes of the GAL pathway, and growth on Galactose as a resulting phenotype.
The authors convincingly demonstrate that different groups of yeast species with distinct ecologies display significant differences in codon optimization in the three GAL genes. The statistical analyses are very well conducted, and notably account for phylogenetic non-independence and multiple testing. While I generally agree with the detailed conclusions of the authors, I am more sceptical regarding the central claim of this manuscript, as reflected in the title. That different species with contrasting ecologies show significant differences in codon optimization does not necessarily implies that the latter allows to predict the former, as this intrinsically depends on the variance of the predictor (here codon usage optimization). As such, the presented results do not strictly assess how ecological variables can be predicted from the genomic pattern of codon usage. Such an approach would require at least some kind of linear modelling allowing to quantify the prediction error and some cross-validation approach to assess how accurate such prediction would be. My guess is (but I may, of course, be wrong) that this accuracy would be rather low, given the large variance in the correlation between codon usage and growth (Figure 2), as well as the overlap between the distributions of optimal codon usage between species groups (figure 3). On a side note, I would say that the results presented here, at best, show that growth on Galactose can be predicted from codon usage. While these results potentially can constitute a proof-of-concept for other studies, it might still be a strong take to claim that such methods can predict metabolic ecology in general.

We thank the reviewer for these comments. We have tempered the language throughout the revised manuscript, including the title, to tone down our approach's ability to predict metabolic ecology and to stress that this is a proof-of-concept analysis that will need to be expanded to include additional predictive measures.

we have added a predictive analysis of two new organisms, Kluyveromyces wickerhamii (Saccharomycetaceae) and Wickerhamiella occidentalis (Dipodascaceae/Trichomonascaceae clade). By characterizing the codon optimization of the GALactose metabolism genes of the two species' genomes, we used a phylogenetically-corrected regression analysis inferred on the data of all previously characterized species to predict each species' growth rate on galactose. To experimentally test our predictions, we measured quantitative growth rate in galactose media of both strains in the laboratory. In K. wickerhamii, we predicted growth rates of 0.65, 0.47, and 0.44 based on GAL1, GAL7, and GAL10 sequences, respectively. The growth rate measured in the laboratory was 0.58. In W. occidentalis, we predicted growth rates of 0.53, 0.76, and 0.54 based on GAL1, GAL7, and GAL10 sequences, respectively. The growth rate of W. occidentalis measured in the laboratory was 0.86.

To better understand the performance of our growth rate predictions based on codon usage, we tested how well GALactose genes with randomly assigned codons could predict growth rate. For each GAL gene associated with K. wickerhamii and W. occidentalis, we generated 1,000 DNA sequences with random codon usage (while keeping the protein sequence identical). For each random sample, we then predicted growth rate based on our regression analysis. In both cases, both the predicted and the actual growth rates fall outside of the 95th percentile (see figure below). Additionally, for all but one observation (GAL7 in K. wickerhamii) the predictions and actual growth rate fall outside the 99th percentile. These results show that our predictions are highly informative.

While neither estimate is perfect, these results suggest that the simple phylogenetic linear model we constructed based on codon optimization of a pathway's gene has predictive power for growth phenotypes. It is likely that a more sophisticated model using more genomes and multiple genes within the major clades would provide even more accurate estimates. Furthermore, K. wickerhamii belongs to the Saccharomycetaceae clade in which we have made ecological predictions. The optimization of K. wickerhamii GAL genes lie below the lower quantile of species found in dairy-associated niches: GAL1 below 0.892 (observed 0.887), GAL10 below 0.843 (observed 0.660), and GAL7 below 0.776 (observed 0.531). We would therefore predict that K. wickerhamii is not associated with dairy niches. Indeed, examination of the literature suggests that K. wickerhamii is "confined to Drosophila species and to tree exudates that probably act as Drosophila habitats" (1. Kurtzman, C.P., Fell JW. The yeasts a taxonomic study 5th edn. Elsevier Science Publishers, Amsterdam. The Yeasts. 2011.)

These results and figure are now featured in section "Can we predict growth rate on galactose from GAL codon optimization data?" from lines 457 to 475 of the revised manuscript.

Minor:
l181+: it would be useful to mention how tRNA quantities were estimated. Is it based on tRNA annotations in the genome (gene copy numbers), or from the sequencing of the tRNA pool in the cell?

All tRNA quantities were estimated based on gene copy numbers. We believe this represents a valid baseline for expression of tRNAs over evolutionary time. Furthermore, measuring the steady state tRNA levels for 300+ species is both cost- and time-prohibitive.

l250: As far as I'm aware of, the ade4 package does not contain PIC of PGLS methods. Do the authors mean the 'ape' package?

We have corrected the text to include the correct package on line 272

l370: the PGM genes have not been introduced before. As I understand, these are used as a negative control... a sentence to clarify this would be helpful.

We have clarified the introduction of the *PGM* genes on lines 434-439**.**

l490: randomly chosen VPS4 gene. A more rigorous approach would consist in defining a statistic based on the branch lengths, and compare the value of the GAL genes to the empirical distribution obtained by computing the statistic on a random sample of genes (rather than taking a single gene).

We agree with the reviewer's suggestion. We have added an analysis comparing the branch length associated with synonymous substitutions in the *GAL* genes with the distribution of branch lengths of 651 BUSCO genes with confidence intervals <10 for all branches on the tree. This new analysis supported the conclusion that synonymous substitutions are more constrained in the two *Kluyveromyces* species isolated from dairy. In *K. aestuarii* and *K. marxianus*, the *GAL* genes all fell within the median 50% of BUSCO genes observed, suggesting that the synonymous sites in these species have not experienced rapid change or high levels of conservation. In the sister species *K. dobzhanskii* and *K. lactis*, there was some evidence of relaxation of selection on synonymous sites in *GAL10* and *GAL1* in *K. dobzhanskii*, leading to longer branches. In *K. lactis*, *GAL7* also had a short branch that fell within the most slowly evolving 25% of the highly conserved BUSCO genes, which may indicate constraint on the codon usage within this gene. This finding is consistent with the strong association between *GAL7* and growth rate on galactose-containing medium.

We have substituted this analysis for the trees previously found in Figure 4C.

l512-514: "PIC correlation implies adaptation". I think this is a bit of a strong statement. This analysis shows that the correlation is not due to phylogeny only. But as the authors mention

further in the manuscript, this could be due to a spurious correlation with another variable (for instance, shared metabolic intermediates). The PIC analyses do not preclude this possibility.

This line was intended to illustrate that the association was not due to phylogenetic similarity. This section has been substituted for a different analysis and is no longer in the manuscript.
