# Supplementary material for: Signatures of optimal codon usage in metabolic genes inform budding yeast ecology
Source: PLoS Biol. 2021 Apr 19;19(4):e3001185. doi: 10.1371/journal.pbio.3001185 (PMC8084343; doi:10.1371/journal.pbio.3001185)
Supplement: Attachment — Submitted filename: GAL_Codon_final_response_reviewers.docx [file pbio.3001185.s018.docx]

Dear Dr Rokas,

Thank you for submitting your revised Research Article entitled "Signatures of optimal codon usage associate metabolic function with ecology in budding yeasts" for publication in PLOS Biology. I have now obtained advice from the original reviewers and have discussed their comments with the Academic Editor.

Based on the reviews, we will probably accept this manuscript for publication, provided you satisfactorily address the remaining points raised by the reviewers. Having discussed these with the Academic Editor, we don't think the experiments suggested by reviewer 2 are essential for publication in our journal, but we would welcome them if you wish to add them to strengthen the study. However, you should include the analysis suggested by reviewer 4, since it is straightforward.

We are excited to hear that our manuscript is likely to be accepted and thank you for your guidance on how to deal with the reviewers’ remaining comments. To address these comments, we have included the additive codon optimization models suggested by reviewer 4. We have not, however, added confidence intervals to our phylogenetic generalized least squares regression as this process is not straightforward due to the influence of phylogeny on the regression.

We also want to suggest a change in your title, to "Signatures of optimal codon usage inform about the metabolic ecology of budding yeasts"

We agree with the reviewers that a change in title is helpful. We, however, are hesitant to include the term “metabolic ecology” in the title as this term is often used as shorthand for the “Metabolic Theory of Ecology” or MTE. MTE refers specifically to the relationship between metabolic rate and ecology. Therefore, we propose this alternative title:

“Signatures of optimal codon usage in metabolic genes inform budding yeast ecology”

Would that work? We are open to any other alternatives that don’t include the phrase “metabolic ecology”.

Please also make sure to address the data and other policy-related requests listed below my signature.

DATA POLICY:

We note that you have provided all your sequence analyses in Figshare repositories or in the supplementary files. In addition, we need you to provide all individual quantitative observations that underlie the data summarized in the figures and results of your paper.

Regardless of the method selected, please ensure that you provide the individual numerical values that underlie the summary data displayed in the following figure panels: Figures 2A-C, 3AB, and 4BC.

NOTE: the numerical data provided should include all replicates AND the way in which the plotted mean and errors were derived (it should not present only the mean/average values).

Please also ensure that each figure legend in your manuscript includes information on where the underlying data can be found and that your supplemental data file/s has/have a legend.

Please ensure that your Data Statement in the submission system accurately describes where your data can be found.

We have added an additional supplementary information excel sheet that contains the raw numerical values used to create Figures 2A-C, 3A-B, and 4B-C. This information is now referenced in the appropriate figure legends. We have also ensured that the Data Statement is up to date and describes where all the data can be found.

As you address these items, please take this last chance to review your reference list to ensure that it is complete and correct. If you have cited papers that have been retracted, please include the rationale for doing so in the manuscript text, or remove these references and replace them with relevant current references. Any changes to the reference list should be mentioned in the cover letter that accompanies your revised manuscript.

We have not made any changes to the list of papers cited in our manuscript.

We expect to receive your revised manuscript within two weeks.

Reviewer remarks:

**Reviewer #1:** The authors have made substantial efforts to address the concerns of the reviewers. The most important revisions include:

1. Changes to the title and some text that tones down the claims to predictive power 2.Including a description of the difference in growth rate on galactose and GAL codon optimization in S. cerevisiae and S. uvarum 3. Attempting to experimentally test the power of the predictive power using K. wickerhamii and W. occidentalis. It should be noted that this was not entirely successful. It might have been more convincing to test more species, or to test species with larger differences between the predicted the predicted growth rates.

We appreciate the reviewer’s acknowledgement of the major changes made. We agree that testing of additional species would be useful – the species included were chosen based on limiting factors such as availability in the lab and presence of selection on codon usage. Current sequencing endeavors of all 1,000+ known species of budding yeasts will allow for additional testing in the future. We have included this point on lines 304-305

4. Demonstrating that GAL gene optimization is not correlated with growth on mannose, and that codon optimization of a mannose metabolism gene PMI40 is not correlated with growth on galactose. The latter point is not particularly convincing however because codon optimization of PMI40 is not correlated with growth rate on mannose either, and was possibly not the best choice to test.

Growth on mannose was chosen primarily because of prior knowledge that a single gene, *PMI40*, is associated with mannose metabolism. As the reviewer rightly notes, *PMI40* optimization is not associated with growth, suggesting that this gene may play additional roles in the cell (other than in carbon metabolism). Future work on this gene is needed to more completely understand why this gene is optimized across species and ecologies. While it would have been nice if *PMI40* was correlated with growth on mannose, we believe that this example nicely illustrates some of the limitations of using codon usage optimization of metabolic genes for inferring ecology. As we noted in the revised manuscript, “This suggests […] that optimization in some pathways may better correlate with growth rate than in others”.

5. Including a more detailed description of genes in other KEGG pathways that correlated with optimization of GAL genes.

We thank the reviewer for their insight on the KEGG pathways.

I do not have sufficient expertise to comment on how important the study is to the general area of ecological prediction. However, in my opinion the data is strong and the analysis is interesting.

We thank the reviewer for their feedback. We believe that this work expands our ability to understand ecology from the genome by adding an additional layer of information on top of standard types of analyses, such as examinations of gene presence-absence.

**Reviewer #2:** I appreciated the additional analyses performed by the authors to adress previous comments on their manuscript. Although more limited than experimental testing, this provides support to the conclusions and interpretations of this work, and clarified several ambiguous points from the previous version. Following previous recommendations and comments, I will be very much looking forward to thorough experimental testing of this work in follow up studies. In the meantime, I think that two points still require attention in this revised version:

We thank the reviewer for their constructive feedback.

1.

"the correlation between GALactose metabolism genes and that growth rate on galactose is specific to this pathway" (L535) - Is this really the case? This seems to contradict the idea that "codon optimization is a valuable genomic feature for linking metabolic pathways with ecological niches" (L149). Is galactose a special case for which codon optimization correlates well with growth rate on the processed substrate?

We have clarified the language noted on L535 to indicate that the optimization in the GAL pathway is specifically associated with growth rate on galactose (as opposed to mannose). Whether galactose is a special or general case of our ability to predict quantitative growth rate from individual gene codon usage optimization will require testing of additional metabolic genes, pathways, and traits.

(L534) "This suggests not only that PMI40 is highly expressed regardless of metabolic state, but also that optimization in some pathways may better correlate with growth rate than in others". Could this hypothesis be tested quickly in a few selected conditions by Q-RT-PCR?

We agree that Q-RT-PCR could provide additional insights into this analysis. This work, however, does not fit within the scope of this revision.

2.

I found the "Prediction of Growth Rate from Unsampled Genomes" section a valuable addition to the revised manuscript. "we generated 1,000 coding sequences with identical amino acid sequences to the observed GAL protein that had randomly assigned codon usage" (L378), to assess the predictive power of this analysis, it should be conducted on all genomes (codon optimized or not) for which growth rates on galactose are known. How often do efficient galactose users fall within the 95% CI? Reciprocally, how often do poor galactose users have predicted growth values outside the 95%CI?

We conducted the random codon growth prediction for the 94 species for which we had growth data and which were initially used to construct the codon optimization – growth rate correlations. For all gene annotations for each species, we randomly assigned codons for each amino acid 1,000 times. If there were multiple copies of a gene in a species, for each random permutation we took the higher of the two codon optimizations (as we did in the original model). We then used the PGLS analysis to predict growth rate from each codon optimization value. This gave us a distribution of 1,000 growth rates. We then assessed if the growth rate measured in the laboratory fell within the 95^th^ and 99^th^ confidence intervals.

Out of 282 predictions (3 *GAL* genes * 94 species = 282 predictions), 39 (14%) fall within the 95^th^ confidence interval. Falling within the confidence interval suggests that we would be unable to distinguish the predicted codon usage from random versus optimized codon usage. The remaining 243 predictions were significant.

All of the genes preformed at nearly the same level with 11 (12%), 16 (17%), and 12 (13%) of the predictions falling within the 95^th^ CI for *GAL1, GAL10,* and *GAL7*, respectively. Two factors seem to play a role in distinguishing random from optimized predicted growth rate: 1) the strength of the growth rate measured in the laboratory, and 2) the amino acid composition of the gene.

First, the higher the growth rate the more likely the true growth rate falls outside of the 95^th^ CI of random codon usage predictions. For all species with growth rates above the median (0.54), all of the measured growth rates fall outside of the 95^th^ CI for *GAL1.* This makes intuitive sense – without a high growth rate, the genes would likely be expressed at low levels and there would be low selective pressure for increased optimization. In such cases, mutational bias and random drift would be expected to give rise to random codon usage. Due to this random codon usage, there is increasing chance of inaccurate prediction at low codon optimization.

Second, the width of the distribution of random codon usage (and its confidence intervals) is influenced by the amino acid content of the gene. There are not only differences in the number of synonymous codons associated with each amino acid between species but also in the weight of each synonymous codon. Therefore the 95^th^ CI have a width ranging from 0.0053 to 0.52. For all predictions (282) that fall below the median width of the 95^th^ CI (aka 95^th^ CI of < 0.129 or 141 observations) only 4 had measured growth that fell within the CI.

While these results are interesting, we believe they are beyond the scope of the current manuscript; thus, we have not added them to the manuscript.

**Reviewer #3:** The authors have addressed my concerns.

We thank the reviewer for all their work on helping us improve this manuscript

**Reviewer #4:** The authors submitted an extensive revision of their manuscript, addressing the comments pointed by the editor. The revised manuscript includes (1) a rewriting of the text and title to better match the study's outcome and (2) new results strengthening the manuscript's central claim. I only have minor comments on the additional analyses, listed below.

We thank the reviewer for their comments, which helped us improve this article.

1) l302+: It is unclear why the PGLS analysis did not need a new tree, compared to PICs. PICs can be seen as a particular case of PGLS, and both depend on an underlying phylogenetic tree. Can the author clarify this point?

A PIC transforms each variable independently and then a linear regression is performed on these independent contrasts. In order to add new data points (independent contrasts) to the linear regression, we would need to phylogenetically transform these points (this requires a phylogenetic tree). On the other hand, A PGLS is a transformation of the regression based on the amount of phylogenetic signal in the data. Because it is the regression (and not the raw data) that is transformed in a PGLS, we can then use new raw data to estimate the growth rate. We have now clarified this point on lines 302 -304.

2) Growth rate prediction: the authors run one model per gene and obtain three growth rate estimates, one per gal gene. Would it be possible (and make sense) to combine data of the three genes to get a single estimate? A confidence interval of the estimates could be provided, either by maximum likelihood from the PGLS prediction or by means of a bootstrap approach, resampling codon sites in all genes.

We had previously tested multiple additive models for growth rate prediction but found that they decreased the correlation between growth rate and codon optimization than *GAL7* alone. Compared to *GAL7* alone (p-value of 3.21e-09), the second-best model included *GAL7* and *GAL10* (growth rate ~ *GAL7 + GAL10*) and had a p-value of 1.82e-08. This supports our previous finding that *GAL7* has the most predictive power for growth rate. We have now included these analyses on lines 429-431. Confidence intervals around a phylogenetic generalized least squares regression are influenced by the phylogenetic relatedness of the species and their estimation is not straightforward.

3) Figure 4. B: for K. lactis, it is unclear where the 75% quantile line is. Maybe an arrow on the x-axis would be more precise if the dashed line is too close to the gene values? (alternatively, indicate the gene values with arrows and the quantiles with dashed lines.) Quantiles could also be displayed in subfigure 4C, for consistency.

We have updated the figures for clarity and consistency.
